# Upregulated FOXM1 stimulates chondrocyte senescence in Acot12^-/-^Nudt7^-/-^ double knockout mice

**DOI:** 10.7150/thno.89033

**Published:** 2023-09-25

**Authors:** Jinsoo Song, Ee Hyun Kim, Jun-Ho Yang, Donghyeon Kim, Akhmad Irhas Robby, Se-ah Kim, Sung Young Park, Ji Hyun Ryu, Eun-Jung Jin

**Affiliations:** 1 Department of Biological Sciences, College of Health Sciences, Wonkwang University; Iksan, Chunbuk, 570-749, Korea.; 2 Graduate School of Pharmaceutical Sciences, College of Pharmacy, Ewha Womans University; Seoul 03760, Korea.; 3 Department of Chemical and Biological Engineering, Korea National University of Transportation; Chungju 27469, Korea.; 4 Department of Carbon Convergence Engineering, Wonkwang University; Iksan, Chunbuk, 570-749, Korea.

**Keywords:** osteoarthritis, *Acot12^-/-^Nudt7^-/-^*, * FoxM1*, Rho@PAA-MnO_2_, heparin-ACBP/COS-GA-*siFoxM1*

## Abstract

**Rationale:** One of the hallmarks of osteoarthritis (OA), the most common degenerative joint disease, is increased numbers of senescent chondrocytes. Targeting senescent chondrocytes or signaling mechanisms leading to senescence could be a promising new therapeutic approach for OA treatment. However, understanding the key targets and links between chondrocyte senescence and OA remains unclear.

**Methods:** Senescent chondrocytes were identified from *Nudt7^-/-^, Acot12^-/-^*, double-knockout mice lacking *Acot12* and *Nudt7* (dKO) and applied to microarray. The presence of forkhead transcription factor M1 (FOXM1) was detected in aged, dKO, and destabilization of the medial meniscus (DMM) cartilages and articular chondrocytes, and the effect of *FoxM1* overexpression and acetyl-CoA treatment on cartilage homeostasis was examined using immunohistochemistry, quantitative real-time PCR (qRT-PCR), cell apoptosis and proliferation assay, and safranin O staining. Delivery of Rho@PAA-MnO_2_ (MnO_2_ nanosheet) or heparin-ACBP/COS-GA-*siFoxM1* (ACBP-*siFoxM1*) nanoparticles into DMM cartilage was performed**.**

**Results:** Here, we propose the specific capture of acetyl-CoA with the delivery of (*FoxM1* siRNA (*siFoxM1*) to prevent cartilage degradation by inhibiting the axis of chondrocyte senescence. dKO stimulate chondrocyte senescence via the upregulation of *FoxM1* and contribute to severe cartilage breakdown. We found that the accumulation of acetyl-CoA in the dKO mice may be responsible for the upregulation of *FoxM1* during OA pathogenesis. Moreover, scavenging reactive oxygen species (ROS) induced by chondrocyte senescence via the implantation of MnO_2_ nanosheets or delivery of *siFoxM1* functionalized with acetyl-CoA binding protein (ACBP) to capture acetyl-CoA using an injectable bioactive nanoparticle (*siFoxM1*-ACBP-NP) significantly suppressed DMM-induced cartilage destruction.

**Conclusion:** We found that the loss of *Acot12* and *Nudt7* stimulates chondrocyte senescence via the upregulation of *FoxM1* and accumulation of acetyl-CoA, and the application of *siFoxM1*-ACBP-NP is a potential therapeutic strategy for OA treatment.

## Introduction

Osteoarthritis (OA), the most common progressive chronic joint disease, is characterized by structural modification primarily to articular cartilage and subchondral bone, but also to synovia, ligaments, and muscles, indicating that OA pathogenesis is a complex process that involves and affects the cartilage and entire joint tissues 1. The physiological and pathological changes in the joint tissue during the occurrence and development of OA are mediated by the interplay of various factors involved in maintaining cartilage homeostasis, including proinflammatory cytokines such as interleukin (IL)-1β, tumor necrosis factor (TNF)-α, IL-6, and anti-inflammatory cytokines such as IL-4 and IL-10; growth factors such as transforming growth factor-β and fibroblast growth factor-21; and adipokines 2. An imbalance among these factors within the cartilage impairs the stable turnover of the cartilage matrix and results in cartilage degeneration and destruction. Traditionally, the management of OA has been constrained to relieve the joint pain using the non-steroidal anti-inflammatory drugs (NSAIDs) or analgesics rather than targeting the components of structural disease progression 3. However, since their side-effects often restrict their use, there is an urgent need for new biological targets for intervention and treatments designed to prevent or reverse the symptoms of OA. Given correlation between chondrocyte senescence and OA 4,5, targeting senescent chondrocytes or responsible signaling mechanism could be a novel approach for OA therapy. The impact of senescence on OA is complex and just beginning to be understood. Senescent cartilage cells express high levels of senescence biomarkers such as p53 and the cyclin-dependent kinase inhibitors p21 and p16^INK4a^ 6. Recent study demonstrated rejuvenating effects of exosomes derived from umbilical cord-derived mesenchymal stem cells on senescent OA chondrocytes through modulating p53 signaling pathway 7. Age-related reactive oxygen species (ROS) production of chondrocytes plays a role in cartilage degradation as well as chondrocyte cell death 8. ROS accumulate dysfunctional mitochondria, induce DNA damage, and lead to premature senescence, degradation of matrix and loss of bone mass in OA 9.

Senescent cells showed a high metabolic activity, despite the decline in their proliferative potential. Chondrocytes depend mainly on anaerobic metabolism to develop, maintain, and repair the cartilage matrix. Anaerobic glycolysis is stimulated in OA chondrocytes to generate sufficient ATP for maintaining homeostasis in the cartilage matrix 10,11. Pyruvate kinase M2, a critical enzyme involved in the glycolytic step that converts phosphoenolpyruvate to pyruvate with the generation of ATP, is upregulated in OA chondrocytes compared to healthy chondrocytes 12. Furthermore, in OA tissues, the tricarboxylic acid cycle is also upregulated, most likely increasing the production of ATP required to repair cartilage damage 13-15. The fatty acid β-oxidation pathway, a catabolic process in which fatty acid molecules are broken down to provide energy, is another metabolic pathway that is affected during OA pathogenesis 16,17. Mitochondrial β-oxidation of fatty acids plays an essential role in the development and progression of OA 18,19. Dysregulation of oxidized fatty acid derivatives impairs the homeostasis of the cartilage matrix and reduces cell viability, as observed in osteoarthritic cartilage 16,20. Exposure of human chondrocytes to linoleic acid stimulates the secretion of IL-6 and the production of prostaglandin E2, resulting in biological responses that are closely related to the severity of OA 21. Palmitic acid induces chondrocyte apoptosis, which is associated with the accumulation of lipid droplets 22,23. Accumulated fatty acids in OA cartilage suggest that mitochondrial dysfunction may be closely related to the pathogenesis of OA, as mitochondria use lipids as the main fuel to produce chemical energy that controls many cellular processes; *i.e.* accumulated fatty acids may affect the biological pathways involved in cartilage degradation, including oxidative stress, inflammation, and chondrocyte apoptosis through mitochondrial dysfunction. Recently, we demonstrated a functional interaction between peroxisomes and mitochondria in the regulation of lipid metabolism 24. Furthermore, impaired peroxisomal function in the regulation of fatty acid β-oxidation results in an imbalance in cartilage matrix homeostasis, which is closely associated with the pathogenesis of OA. Peroxisomes are small ubiquitous membrane-bound organelles that play many vital roles for various metabolic pathways, including the β-oxidation of very long chain fatty acids (VLCFA) and LCFA, synthesis of ether lipid, and bile acid metabolism. Among various peroxisomal enzymes, our laboratory recently suggested that nudix hydrolase 7 (NUDT7), which hydrolyzes the diphosphate of coenzyme A (CoA), plays an important role in maintaining cartilage homeostasis 25. Deficiency of *Nudt7* impairs the peroxisomal β-oxidation and accumulates the acetyl-CoA in cartilage leading to the inflammation and apoptotic cell death via activation of phosphoglycerate mutase 1, one of key enzymes in aerobic glycolysis. Moreover, our laboratory showed that in Acyl-CoA thioesterase 12 knockout (*Acot12^-/-^*) cartilage, accumulation of acetyl-CoA stimulates *de novo* lipogenesis and consequently contributes to the degradation of the cartilage matrix 26. These data suggest the importance of peroxisomal β-oxidation of fatty acids, particularly acetyl-CoA, in maintaining cartilage homeostasis during OA pathogenesis. However, the mechanism by which peroxisomal β-oxidation of fatty acids drives metabolic dysfunction in OA cartilage remains unclear. Furthermore, the underlying regulatory and signaling mechanisms remain largely unknown. In the current study, we generated double-knockout mice lacking *Acot12* and *Nudt7* (Acot12^-/-^ Nudt7^-/-^, dKO) to evaluate the regulatory role and signaling mechanism of peroxisomal β-oxidation of fatty acids in the pathogenesis of OA. Our results revealed that dKO mice spontaneously developed severe degradation of the cartilage matrix due to an increased level of cellular senescence via upregulation of forkhead transcription factor M1 (FOXM1) via accumulation of acetyl-CoA. Recently, targeted biomaterials specific to cartilage to synovium have been applied to improve the therapeutic efficiency for OA treatment. Functional biomaterials such as hydrogels, non-hydrogel polymers, and inorganic nanomaterials are widely used for OA delivery, chondrocyte encapsulation, and cartilage engineering 27-29. Most commonly used hydrogels in OA treatment are polysaccharides (*e.g.* chitosan, hyaluronic acid, heparin) and proteins (*e.g.* collagens, gelatin). The advantage of using these biomaterial-based hydrogels is that they overcome the limitation of poor drug retention in knee joints. This increases the therapeutic effectiveness by allowing the drug to stay in the joint or cartilage longer and be released slowly. In the current study, we generated the delivery system targeting senescent chondrocytes using biomaterials such as chitosan and heparin using *siFoxM1* and acetyl-CoA binding protein (ACBP) and evaluated the therapeutic effect.

## Results

### Loss of Acot12 and Nudt7 involves OA pathogenesis

**Figure 1:**
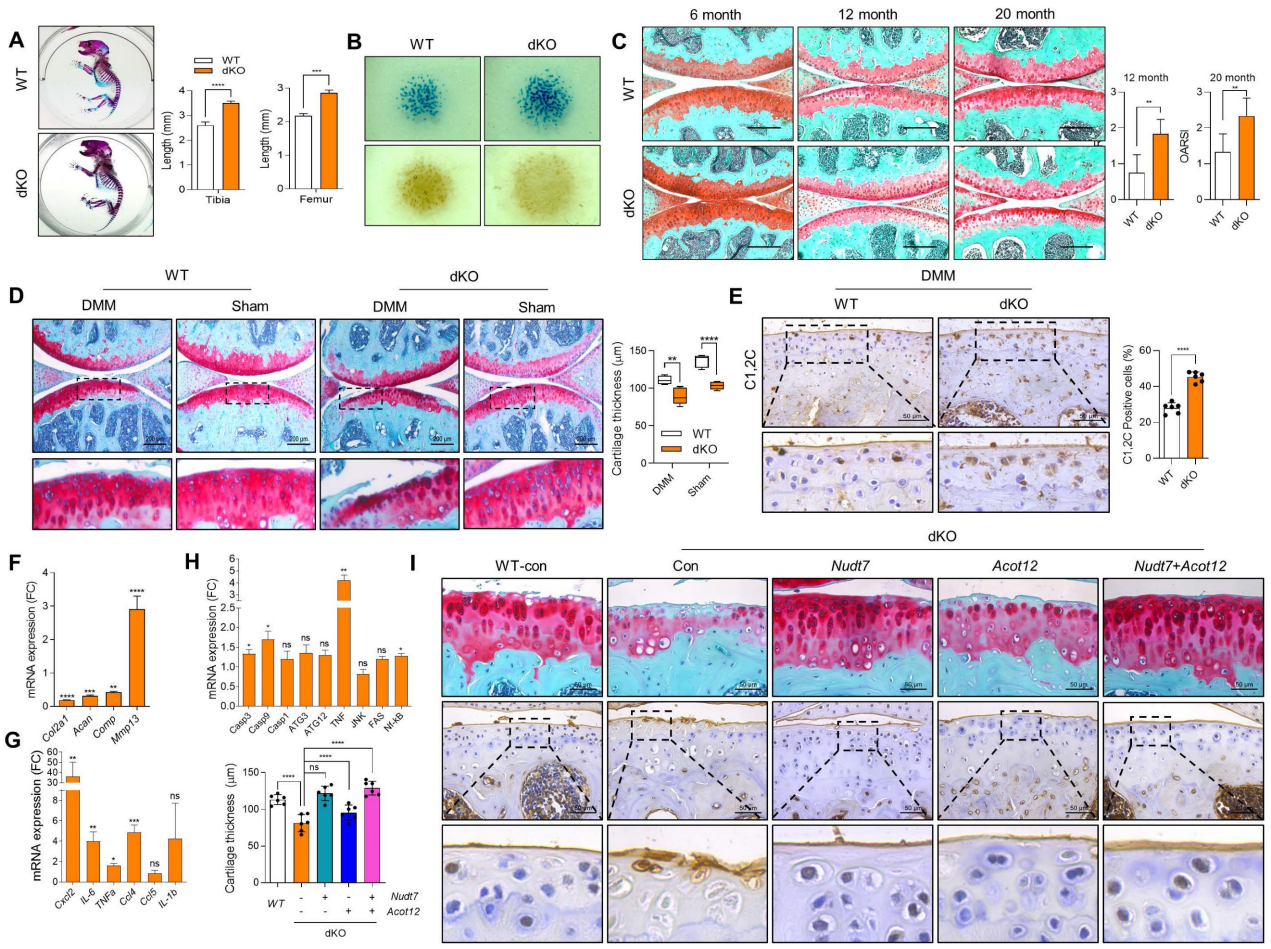
** Musculoskeletal and cartilage homeostasis in dKO mice. (A)** Alizarin red/Alcian blue staining of WT and dKO newborn mice (n = 4 per group). **(B)** PNA and Alcian blue staining of micromass cultures of WT and dKO limb mesenchymal cells. **(C)** Safranin O staining of cartilage of 6-, 12-, and 20-month-old WT and dKO mice. The degree of cartilage degradation is quantified according to the OARSI grade. Scale bar = 200 μm. **(D)** Safranin O staining of DMM cartilage of WT and dKO mice. Cartilage thickness was measured (n = 5 per group). Scale bar = 200 μm. **(E)** Immunohistochemistry of C1,2C in WT and dKO DMM cartilage at eight weeks post-surgery (n = 4 per group). **(F-H)** Expression level of catabolic and anabolic genes in maintaining cartilage homeostasis with WT and dKO DMM cartilage (n = 3 per group). **(I)** Safranin O staining and immunohistochemistry of C1,2C in WT, or dKO cartilage with or without restoration of *lenti-HA-Nudt7, -Acot12*, or both. cartilage thickness was measured. Scale bar = 50 μm. *P < 0.05; **P < 0.01; *** P < 0.001; **** P < 0.0001.

We previously reported that two peroxisomal enzymes, *Acot12* and *Nudt7* are important for maintaining cartilage homeostasis 25,26. Moreover, the accumulation of acetyl-CoA due to impaired β-oxidation of LCFA and VLCFA in *Acot12^-/-^* - or *Nudt7^-/-^* cartilage could be a key player in the regulation of cartilage homeostasis through the modulation of catabolic and inflammatory responses (Figure S1A). We generated *Acot12^-/-^Nudt7^-/-^* mice (Figure S1B). Unlike our previous reports demonstrating no difference in the skeletal development of *Acot12^-/-^* 26 or *Nudt7^-/-^* mice 25, skeletal development in dKO mice, that is, the length of the tibia and femur of postnatal mice and endochondral ossification, was significantly altered (Figure 1A). Moreover, micromass cultures of dKO mouse limb bud mesenchymal cells showed significantly increased cellular condensation and chondrogenic differentiation, as assessed by Peanut agglutinin (PNA)- and Alcian blue staining, respectively, compared to *Acot12^+/+^Nudt7^+/+^* (wild-type, WT) mesenchymal cells (Figure 1B). Severe cartilage degradation was observed in 12- and 20-month-old dKO mice compared to WT mice (Figure 1C). Tibialis anterior (TA) muscle mass, analyzed by measuring cross-sectional area, also significantly decreased with dysregulation of peroxisomal and mitochondrial gene profiles in 12- and 20-month-old dKO mice (Figure S2). These data suggest that *Acot12^-/-^ Nudt7^-/-^* dKO exacerbates the degeneration of the musculoskeletal system, such as cartilage and muscle, possibly by functional interaction with peroxisomes and mitochondria.

To investigate the effect of dKO on the pathogenesis of OA, destabilization of the medial meniscus (DMM) was performed to induce OA pathological conditions. Severe cartilage degradation, as assessed by safranin O staining, was observed in DMM-induced dKO cartilage compared to control cartilage (Figure 1D). A dramatic reduction in cartilage thickness was also observed in the DMM-induced dKO cartilage. The intensity and number of Collagen C1,2C positive cells in DMM-induced dKO cartilage were significantly increased compared to that in WT cartilage (Figure 1E). Moreover, the transcription levels of cartilage matrix genes such as *Col2a1, Acan*, and *Comp* decreased (Figure 1F), whereas the expression levels of a major cartilage degrading enzyme, *Mmp13*, inflammatory chemokines and cytokines such as *Cxcl2*, *IL-6*, *TNFα*, and *Ccl4*, and apoptotic genes such as *Casp3* and *Casp9* significantly increased in DMM-induced dKO cartilage (Figure 1G-H). To determine whether restoration of either *Acot12* or *Nudt7* in dKO cartilage could prevent cartilage degradation, *Nudt7*- (*Lenti-HA-Nudt7*) or *Acot12*-encoding lentiviruses (*Lenti-HA-Acot12*) were introduced into the knee joint cavity of dKO mice (Figure 1I). Intra-articular injection of *Lenti-HA-Acot12* or *-Nudt7* significantly reduced cartilage degradation, and a dramatic reduction in cartilage degradation was induced by the co-injection of *Lenti-HA-Acot12* and *-Nudt7*. As observed in *Nudt7^-/-^* and *Acot12^-/-^* immature murine articular chondrocytes (iMACs), cell proliferation significantly decreased (Figure S3 and Figure S4A), and increased apoptosis (Figure S4B) and dysregulation of mitochondrial dynamics (Figure S4C) were observed in the dKO iMACs. However, in dKO iMACs, we did not observe significant lipid accumulation compared to that in WT iMACs, as seen in *Nudt7^-/-^* or *Acot12^-/-^* iMACs (Figure S4D-E). The expression levels of genes involved in lipid metabolism, including *Fasn*, *Scd1*, and *Elovs*, were also decreased in dKO iMACs, whereas they were increased in either *Nudt7^-/-^* or *Acot12^-/-^* iMACs (Figure S4F).

### Loss of Acot12 and Nudt7 significantly stimulates chondrocyte senescence via FoxM1 upregulation

**Figure 2:**
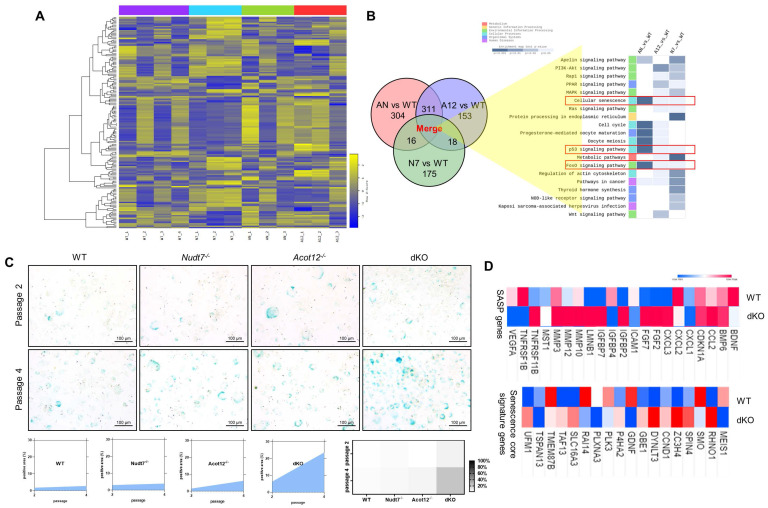
** Chondrocyte senescence in dKO cartilage. (A)** Heatmap analysis of microarray data from WT, *Nudt7^-/-^, Acot12^-/-^*, and dKO iMACs. **(B)** Common pathways analysis of microarray data. **(C)** SA-β-gal staining of passage 2 and passage 4 iMACs isolated from WT, *Nudt7^-/-^, Acot12^-/-^*, and dKO embryos. **(D)** Transcriptional level of SASP and senescence score signature genes in WT and dKO cartilage.

**Figure 3:**
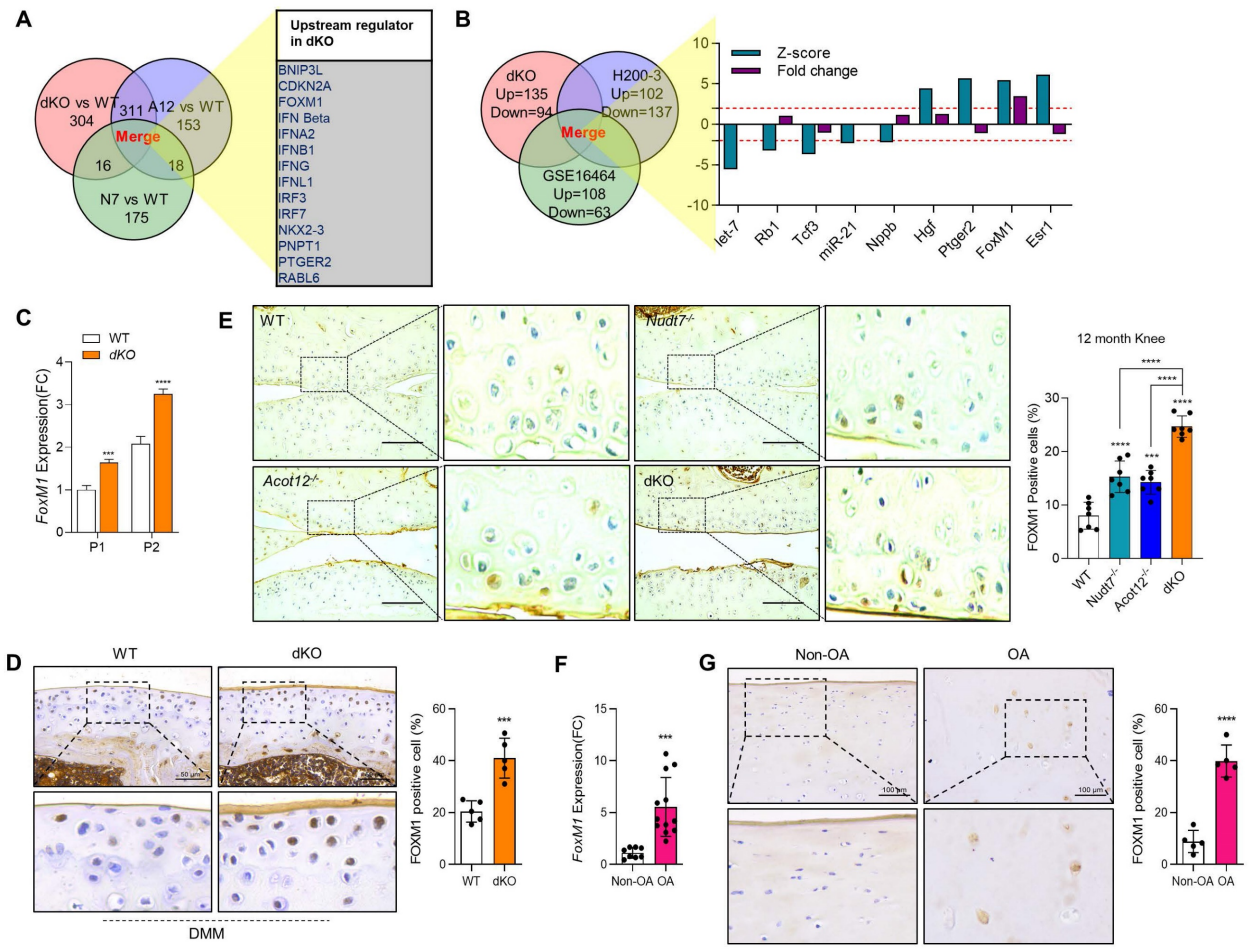
** FoxM1 in aged, dKO, and DMM cartilages**. **(A)** Ven diagram of significantly altered genes from microarray data of *Nudt7^-/-^, Acot12^-/-^*, and dKO iMACs compared to WT iMACs. **(B)** Ven diagram of significantly altered genes from microarray data of dKO iMACs compared to those from human OA patent sequencing data. **(C)** Transcriptional level of *FoxM1* in passaged (P1, P2) WT and dKO iMACs (n = 3 per group).** (D)** Immunohistochemistry of FOXM1 in DMM cartilage of WT and dKO mice (n = 5). FOXM1-positive cell numbers are indicated by dot graph. **(E)** Immunohistochemistry of FOXM1 in 12-month-old WT, *Nudt7^-/-^, Acot12^-/-^*, and dKO cartilage (n = 4 per group). FOXM1-positive cell number were indicated by bar-dot graph. **(F)** Transcriptional level of *FoxM1* in normal and OA patient chondrocytes (n = 8 in normal, n = 12 in OA). **(G)** Immunohistochemistry of FOXM1 in normal and OA patient cartilage (n = 5 per group). FOXM1-positive cell numbers were indicated by dot graph. *** P < 0.001; **** P < 0.0001.

To identify the factors responsible for cartilage degradation modulated by dKO, we performed microarray analysis using iMACs isolated from WT, *Nudt7^-/-^*-, *Acot12^-/-^*-, and dKO iMACs mice, extracted common genes whose expression was significantly altered in *Nudt7^-/-^*-, *Acot12^-/-^*-, and dKO iMACs compared to WT iMACs, and analyzed the enriched signaling pathways (Figure 2A-B). Cellular senescence, the p53 signaling pathway, and the FoxO signaling pathway were suggested as common enriched signaling pathways, with the highest significance in dKO iMACs. *In vitro* analysis of passaging (P2, P4) iMACs showed an increased number of senescence-associated β-galactosidase (SA-β-gal)-positive cells in *Nudt7^-/-^*-, *Acot12^-/-^*-, and dKO iMACs compared to WT iMACs (Figure 2C). The most dramatic increase in the number of senescent cells was observed in dKO iMACs. Moreover, the transcription levels of senescence-associated secretory phenotype (SASP) and senescence core signature genes were also significantly upregulated in dKO iMACs compared to those in WT iMACs (Figure 2D). Gene profile analysis suggested that cell cycle: G2M DNA damage checkpoint regulation, ATM signaling, and cell cycle control of chromosomal replication were the top canonical pathways in dKO iMACs (Figure S5). According to the microarray analysis of common genes whose expression was significantly altered in Nudt7^-/-^-, Acot12^-/-^-, and dKO iMACs compared to WT iMACs, *Bnip3l*, *Cdkn2a*, *Ifn*, *Irf3*, *Irf7*, *FoxM1*, *Nkk2.3*, *Pnpt1*, *Ptger2*, and *Rabl6* were suggested as possible upstream regulators (Figure 3A). Furthermore, we extracted the common genes whose expressions were significantly altered in the microarray of dKO iMACs (up = 135, down = 94), RNA sequencing of OA chondrocytes isolated from OA patients (up = 102, down = 137), and GSE16464 [3D culture of normal vs. OA chondrocytes (up = 108, down = 63)], and identified *Rb1*, *Tcf3*, *Nppb*, *Hgf*, *Ptger2*, *FoxM1*, and *Esr1* (Figure 3B and Figure S5). *FoxM1* was commonly altered in *Nudt7^-/-^*, *Acot12^-/-^*, and dKO iMACs as well as in OA chondrocytes. The expression levels of *FoxM1* increased significantly as passaging progressed, with higher expression levels in dKO iMACs than in WT iMACs (Figure 3C). We also confirmed the upregulated expression level of FOXM1 in DMM-induced dKO cartilage compared to that in DMM-induced WT cartilage (Figure 3D). Compared to *Nudt7^-/-^*or *Acot12^-/-^* cartilage, as well as to WT cartilage, a significantly increased number of FOXM1-positive cells was observed in dKO cartilage (Figure 3E), and the introduction of either *lenti-HA-Acot12* or *-Nudt7* into dKO cartilage reduced these increased FOXM1-positive cells (Figure S6). The expression level of *FoxM1* was also significantly upregulated in patients with OA (Figure 3F). Immunohistochemical analysis showed that the number of FOXM1-positive cells in the OA area of OA cartilage was increased by up to three-fold compared to that in the non-OA area of OA cartilage (Figure 3G).

**Figure 4:**
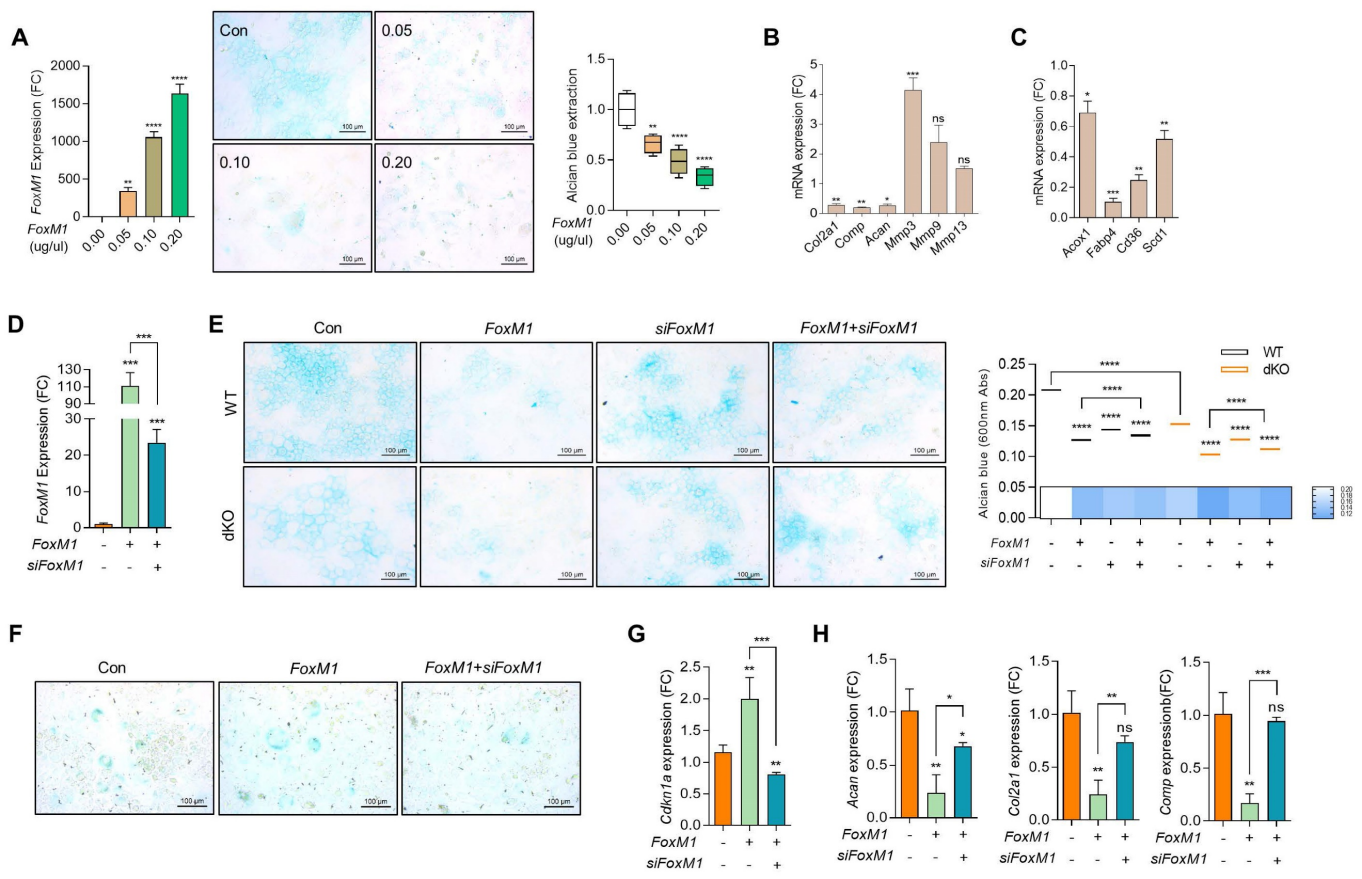
** FoxM1 stimulates chondrocyte senescence. (A)** RT-PCR with dose-dependent introduction of *pcDNA-FoxM1* (left panel), Alcian blue staining (middle panel), Quantification of Alcian blue staining (right panel) (n = 4 per group). *FoxM1* were introduced into iMACs. **(B)** Transcriptional levels of cartilage matrix and cartilage degrading genes. **(C)** Transcriptional levels of genes in lipid metabolism in *FoxM1*-overexpressed iMACs (n = 3 per group). **(D)** Transcriptional level of *FoxM1* (n = 3 per group). **(E)** Alcian blue staining in WT and dKO iMACs transfected with *FoxM1, siFoxM1*, or both (n = 4 per group). Quantification of Alcian blue staining extracted with 6M Guanidine-HCl was measured at a 650 nm absorbance. **(F)** SA-β-gal staining (n = 3 per group). **G)** Expression level of *Cdkn1a* (n = 4 per group).** (H)** Expression level of cartilage matrix genes (n = 3 per group).

To investigate the role and function of FOXM1 in OA pathogenesis, the expression level of FOXM1 was modulated exogenously using *FoxM1*-encoding pcDNA construct (*FoxM1*; Figure S7). As observed in Nudt7^-/-^-, Acot12^-/-^-, and dKO iMACs, the expression levels of mitochondrial genes such as *Cox2* and *Cox4*, peroxisomal genes such as *Pmp70* and *Cat*, and lysosomal genes such as *Lamp1* and *Lamp2* were significantly suppressed by the introduction of *FoxM1* into WT iMACs (Figure S8). The number of Alcian blue-positive cells (Figure 4A) and the expression levels of cartilage matrix genes such as *Col2a1*, *Comp*, and *Acan* were significantly decreased, whereas the expression levels of genes involved in cartilage degradation such as *Mmp-3*, *-9*, and *-13* were significantly increased with the introduction of *FoxM1* into WT iMACs (Figure 4B). Interestingly, as shown in dKO cartilage in Figure S4F, the expression levels of genes involved in lipid metabolism such as *Fabp4*, *Cd36*, and *Scd1* were significantly decreased in *FoxM1*-introduced WT iMACs (Figure 4C). To determine whether small interfering RNA specific to *Foxm1* (*siFoxm1*) could overcome this inhibitory action on cartilage homeostasis, three different *siFoxM1* were introduced into dKO iMACs and their efficiency was examined (Figure S9, Figure 4D). In this study, a significant recovery in the number of Alcian blue-positive cells was observed by the introduction of *siFoxM1* into *FoxM1*-introduced WT or dKO iMACs (Figure 4E left panel). Extraction and photometric quantification of Alcian blue staining (Figure 4E right upper panel) and Image J analysis of the Alcian blue-stained area (Figure 4E right lower panel) were also increased by the introduction of *siFoxM1* into dKO iMACs. Consistent with the top canonical pathway observed in the dKO iMACs (Figure S5), the number of SA-β-gal-positive cells (Figure 4F) and expression level of *Cdkn1a* (Figure 4G) was increased by the introduction of *FoxM1* into dKO iMACs. Decreased levels of cartilage matrix genes such as *Col2a*, *Comp*, and *Acan* by the introduction of *FoxM1* were restored by the co-introduction of *FoxM1*-specific siRNA (Figure 4H).

**Figure 5:**
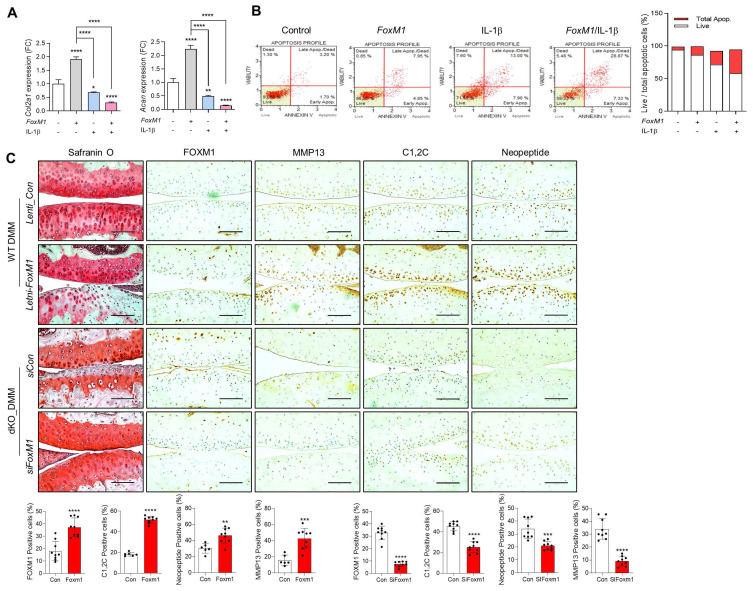
** FoxM1 overexpression exacerbates cartilage degradation. (A)** Transcriptional level of *Col2a1* and *Acan* with introduction of *FoxM1* in the absence or presence of IL-β (n = 3 per group). **(B)** Apoptotic cell death with introduction of *FoxM1* in the absence or presence of IL-β (n = 3 per group). **(C)** Safranin-O staining and immunohistochemistry of FOXM1, MMP13, C1,2C, and Neopeptide in DMM cartilage of WT and dKO mice infected with *FoxM1*- or *siFoxM1*-lentivirus (n = 6-9 per group). Positive cell numbers are indicated by bar-dot graph. *P < 0.05; **P < 0.01; *** P < 0.001.

Next, we exposed WT iMACs into IL-1β, one of the key cytokines involved in OA pathogenesis, to make an OA environment. Exposure of IL-1β into iMACs showed typical OA characteristics, that is, significantly decreased expression levels of cartilage matrix genes such as *Col2a1* and *Acan* (Figure 5A) and increased chondrocyte apoptosis (Figure 5B). These alterations were exacerbated by co-treatment of* FoxM1* in the presence of IL-1β. *In vivo* intra-articular injection of *lenti-HA-FoxM1* into the cartilage of DMM-induced WT mice stimulated cartilage degradation with increased numbers of FOXM1, C1,2C, and MMP13-positive cells (Figure 5C). In contrast, intra-articular injection of *siFoxM1* into the cartilage of DMM-induced dKO mice significantly suppressed cartilage degradation, with decreased numbers of FOXM1, C1,2C, and MMP13-positive cells.

**Figure 6:**
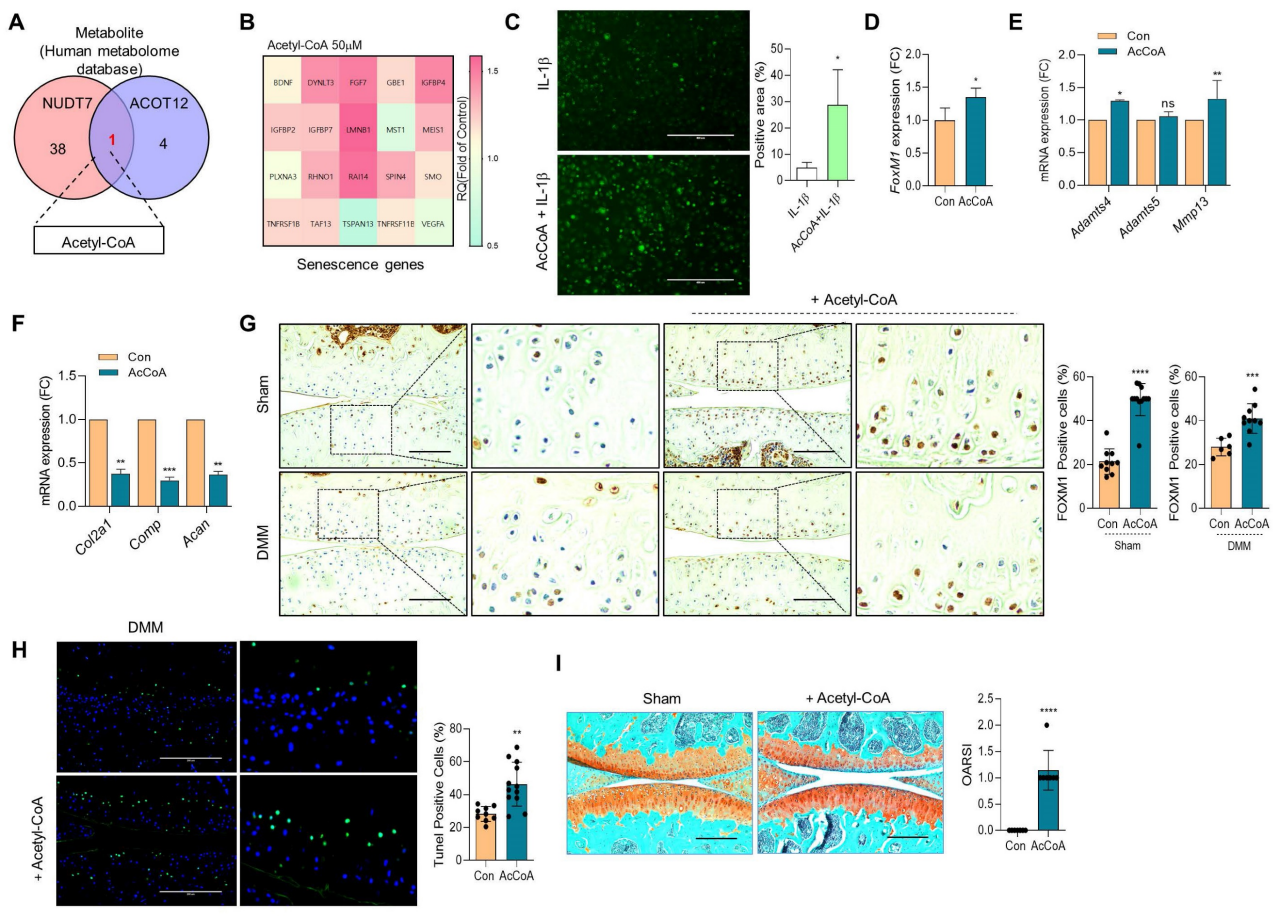
** Acetyl-CoA induces FoxM1 and increases chondrocyte senescence. (A)** Metabolite analysis from human metabolome database. 50 μM Acetyl-CoA were exposed into iMACs. **(B)** Senescence genes signature, **(C)** Senescence-induced ROS level (n = 4), **(D)** Expression level of *FoxM1*, **(E)** Expression level of cartilage degrading genes, **(F)** Expression level of cartilage matrix genes (n = 3). **(G)** Immunohistochemistry of FOXM1 in DMM cartilage with or without introduction of Acetyl-CoA (n = 6-12 per group). FOXM1-postive cell numbers are indicated by the bar dot graph. **(H)** Tunel assay in DMM cartilage with or without introduction of Acetyl-CoA (n = 9-12). **(I)** Safranin-O staining of DMM cartilage with or without introduction of Acetyl-CoA (n = 7). The degree of cartilage degradation is quantified according to OARSI grade. Scale bar = 200 μm. *P < 0.05; **P < 0.01; *** P < 0.001, **** P < 0.0001.

To identify the metabolic molecules underlying chondrocyte senescence and apoptosis induced by dKO mice during OA pathogenesis, we searched the human metabolome database and identified acetyl-CoA as a common metabolite (Figure 6A). To determine whether the elevated level of acetyl-CoA was responsible for FOXM1-induced cellular senescence, iMACs were treated with sodium acetate to accumulate acetyl-CoA (Figure S10). Exposure of iMACs to sodium acetate resulted in typical OA characteristics, that is, decreased *Col2a1* expression and increased *Mmp13* and *Adamts5* expression. Exposure of iMACs to sodium acetate increased the expression levels of senescence signature genes concomitantly with an increase in *FoxM1* levels. In addition, exposure of iMACs to acetyl-CoA also significantly increased the expression of senescence signature genes (Figure 6B), IL-1β-induced ROS (Figure 6C), *FoxM1* (Figure 6D), and cartilage-degrading genes such as *Adamts4* and *Mmp13* (Figure 6E), and decreased the expression levels of cartilage matrix genes such as *Col2a1*, *Comp*, and *Acan* (Figure 6F). Intra-articular injection of acetyl-CoA into the DMM cartilage increased the number of FOXM1- (Figure 6G) and TUNEL- (Figure 6H) positive cells with severe cartilage degradation (Figure 6I).

### In vivo targeting of senescent chondrocytes using heparin-ACBP/COS-GA-*siFoxM1* nanoparticles reduces cartilage degradation

**Figure 7:**
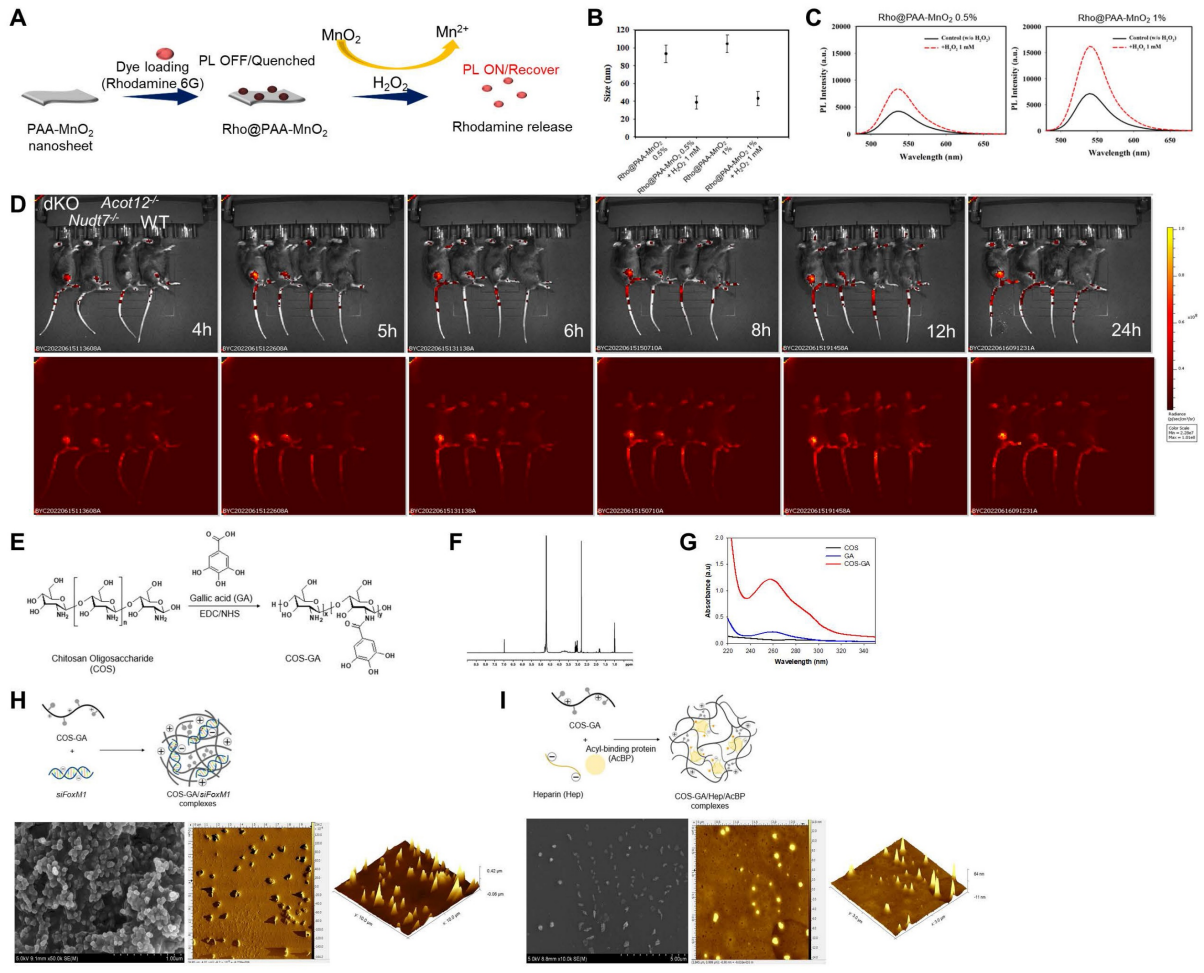
** Cellular senescence detection by Rho-PAA-MnO_2_ sensor. (A)** Scheme of a ROS-responsive fluorescence off-on system with Rho-PAA-MnO_2_ sensor. **(B)** Particle size of Rho@PAA-MnO_2_ (0.5% and 1%) after treatment with 1mM H_2_O_2_ for 30 min obtained by dynamic light scattering spectrometry (DLS). **(C)** Photoluminescence spectra of Rho@PAA-MnO_2_ (0.5% and 1%) after treatment with 1mM H_2_O_2_. **(D)** IVIS images of six-month-old WT, *Nudt7^-/-^, Acot12^-/-^*, and dKO mice implanted with senescence sensor into cartilage. **(E-I)** Preparation and characterizations of COS-GA/*siFoxM1* and COS-GA/Hep/ACBP complexes. **(E)** Synthesis and chemical structures of COS-GA. **(F)** 1H NMR and **(G)** UV-Vis spectra of COS-GA. **(H)** Preparation of COS-GA/*siFoxM1* complexes. SEM (first) and AFM (second and third) images of COS-GA/*siFoxM1* complexes. **(I)** Preparation of COS-GA/Hep/ACBP complexes. SEM (first) and AFM (second and third) images of COS-GA/Hep/ACBP complexes.

To investigate whether cartilage degradation could be inhibited by removing factors that induce chondrocyte senescence in dKO mice, we used a senescence-induced ROS-responsive off-on fluorescence-loaded polyacrylic acid (PAA)-MnO_2_ system (Figure 7A). Fluorescence is quenched by the aggregation of Rhodamine (Rho) and MnO_2_ while it is recovered via the cleavage of MnO_2_ to Mn^2+^ and the release of Rho 5G in the presence of senescence-induced ROS. Rho@PAA-MnO_2_ system was validated by decrease in particle size (Figure 7B) and increase in photoluminescence (PL) intensity after 0.5% or 1% H_2_O_2_ treatment (Figure 7C). Fluorescence recovery with H_2_O_2_ was confirmed by confocal microscopy at 488 nm (Figure S11). *In vitro* analysis of passaged iMACs showed an increase in the intensity of senescence-associated signals as cellular passaging progressed (P4~P9) (Figure S12). Compared with the passaged iMACs of WT mice, the passaged iMACs of *Nudt7^-/-^, Acot12^-/-^,* and dKO mice showed a strong senescence signal. In particular, the strongest senescence signal was detected in passaged iMACs isolated from dKO cartilage. The senescence signal significantly increased in the cartilage of DMM-induced *Nudt7*^-/-^ and dKO mice, with the strongest signal in the DMM-induced dKO cartilage (Figure 7D). Moreover, we confirmed an increase in senescence-associated signals as the OA pathogenesis progressed *in vivo*. The senescence-associated signal increased with degradation of the cartilage matrix over time after DMM surgery (Figure S13). For heparin-ACBP/COS-GA-*siFoxM1* (ACBP-*siFoxM1*) nanoparticles, gallic acid-conjugated chitosan oligosaccharide (COS-GA) was synthesized using standard carbodiimide chemistry. COS was conjugated with GA through the formation of amide linkages between the amine groups of COS and the carboxylic acid groups of GA (Figure 7E). The conjugation of GA to the COS backbone was confirmed by both UV-Vis and UV-Vis spectroscopies. The gallol protons of COS-GA were found in 6.9 to 7.1 ppm in ^1^H NMR spectra (Figure 7F). In addition, an absorbance peak at 261 nm caused by the gallol groups of COS-GA was observed (Figure 7G). As previously reported, the λ_max_ of the absorbance of GA was significantly affected by pH and solvents 30. Thus, COS-GA solutions and standard solutions with different concentrations were prepared in PBS (pH 7.4). The degree of GA substitution in the COS backbone was 14.4% measured by UV-Vis spectroscopic studies. The preparation procedure for COS-GA/*siFoxM1* complexes is illustrated in Figure 7H. The electrostatic interactions between positively charged COS and negatively charged *siFoxM1* were followed by gallol-mediated chemical crosslinking of the COS-GA backbones.

Gallol-containing polymers can be crosslinked in PBS (pH 7.4) by self-oxidation, as previously reported 31. The morphology of the COS-GA/*siFoxM1* complexes after dehydration was examined using scanning electron microscopy (SEM) and atomic force microscopy (AFM). COS-GA/*siFoxM1* complexes were well-dispersed spherical nanoparticles. ACBP-loaded nanoparticles were prepared using COS-GA and heparin (Hep). As shown in Figure 7I, the COS-GA/Hep/ACBP complexes were prepared by polyelectrolyte complexation between COS-GA and Hep. The COS-GA/Hep/ACBP complexes were readily dispersed in PBS (pH 7.4).

**Figure 8:**
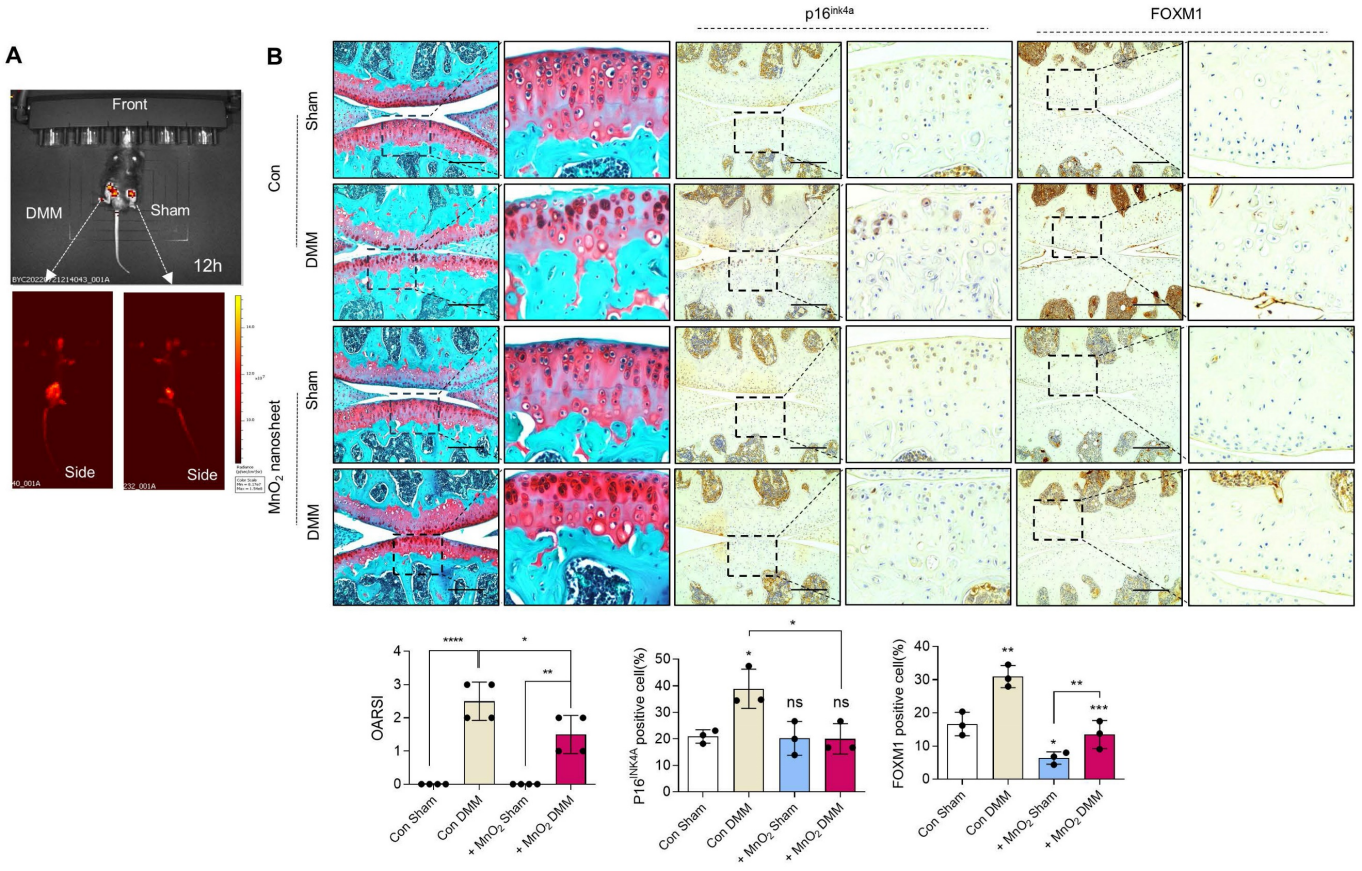
** Delivery of Rho@PAA-MnO_2_ (MnO_2_ nanosheet) significantly reduces cartilage degradation. (A)** IVIS images of sham and DMM cartilage implanted with senescence sensor.** (B)** Safranin-O staining in DMM cartilage implanted with MnO_2_ nanosheet for eight weeks compared to sham cartilage (n = 4 per group). The degree of cartilage degradation is quantified according to the OARSI grade. Scale bar = 200 μm. Immunohistochemistry of P16^ink4a^ and FOXM1 in DMM cartilage (n = 4 per group). Positive cell numbers are indicated by the bar graph. *P < 0.05; **P < 0.01; *** P < 0.001, **** P < 0.0001.

**Figure 9:**
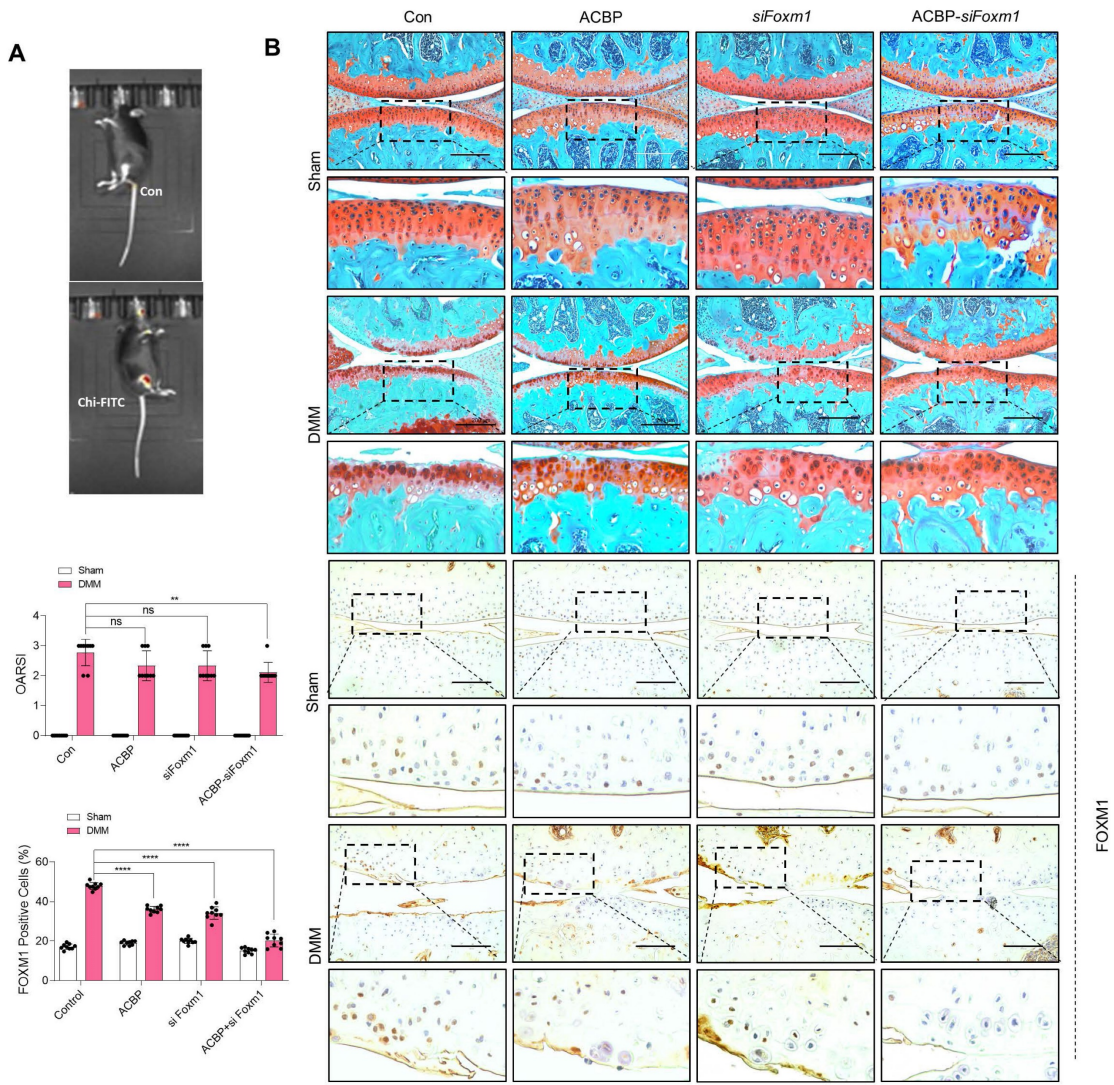
** Delivery of heparin-ACBP/COS-GA-*siFoxM1* (ACBP-*siFoxM1*) nanoparticles significantly reduces cartilage degradation. (A)** IVIS images of DMM cartilage introduced with ACBP-*siFoxM1* nanoparticles compared to sham cartilage.** (B)** Safranin-O staining in DMM cartilage introduced with ACBP-*siFoxM1* nanoparticles for eight weeks compared to sham cartilage (n = 4 per group). The degree of cartilage degradation is quantified according to the OARSI grade. Scale bar = 200 μm. Immunohistochemistry of FOXM1 in DMM cartilage (n = 4 per group). FOXM1-positive-cell numbers are indicated by the bar graph. *P < 0.05; **P < 0.01; *** P < 0.001, **** P < 0.0001.

Implantation of PAA-MnO_2_ nanosheets into the cartilage for eight weeks significantly suppressed DMM-induced cartilage degradation (Figure 8A-B). The expression levels and positive cell numbers of P16^ink4a^ and FOXM1 were significantly decreased by the implantation of the PAA-MnO_2_ nanosheets into the DMM cartilage. Next, to determine whether capturing accumulated acetyl-CoA and FOXM1 from senescent chondrocytes could suppress OA pathogenesis, we applied heparin-ACBP/COS-GA-*siFoxM1* (ACBP-*siFoxM1*) nanoparticles (Figure 9A-B). Introduction of ACBP-*siFoxM1* nanoparticles into the cartilage significantly suppressed DMM-induced cartilage degradation. The most significant inhibition of cartilage degradation was observed after the introduction of the ACBP-*siFoxM1* complex.

## Discussion

Cellular senescence, a state of stable cell cycle arrest, is a controlled process that occurs in diverse biological processes 32. However, the persistence and accumulation of senescent cells can impair diverse cellular functions and induce age-related diseases such as OA. Senescent chondrocytes accumulate with age and increase in number in human OA cartilage compared with normal cartilage 4. Senescent chondrocytes localize near osteoarthritic lesions of the cartilage, but not in intact areas of the cartilage, indicating a positive connection between chondrocyte senescence and OA pathogenesis 4. Chondrocyte senescence is a common molecular mechanism that drives or promotes OA development. SASP, a robust proinflammatory secretome, can cause an imbalance in cartilage homeostasis and alter the structure and function of cells and tissues in the joint 4,33. Expression of the SASP factors, *Mmp-13*, *IL-6*, and *IL-1β* stimulates inflammation and degradation of cartilage, which are the hallmarks of OA. Recently, molecular mechanisms underlying the contribution of cell senescence to the initiation and progression of OA have been reported. Oxidative stress may play a major role in the association between chondrocyte senescence and OA development 34-36. Increased levels of intracellular ROS in the cartilage contribute to aging changes in cells and tissues by causing oxidative damage to proteins, lipids, and DNA, resulting in chondrocyte senescence, apoptosis, and cartilage matrix loss. Furthermore, increased oxidative stress in senescent chondrocytes alters mitochondrial mass, potential, and morphology; accumulates dysfunctional mitochondria; and stimulates inflammatory and matrix catabolic responses in chondrocytes 37,38. However, many other questions, such as other factors that induce chondrocyte senescence or other underlying mechanisms that determine articular degeneration that causes osteoarthritis, remain to be answered. In the present study, we observed a high number of senescent cells with peroxisomal dysfunction. Double-knockout mice lacking *Acot12* and *Nudt7* showed stimulated chondrocyte senescence along with upregulation of inflammatory and cartilage catabolic factors and downregulation of cartilage anabolic factors, resulting in the development and progression of OA pathogenesis.

Deregulation of organelle interactions between the endoplasmic reticulum, mitochondria, endosomes, and cellular metabolites contributes to cellular senescence and is associated with aging 39. Mitochondria are closely involved in cellular senescence 4. Mitochondrial dysfunction/alteration-associated senescence produces the SASP, and mitochondria in senescent hepatocytes have an impaired capacity for fatty acid oxidation, leading to increased hepatic lipid deposition, a typical pathophysiological characteristic of non-alcoholic fatty liver disease 40,41. In this study, we found that the dysfunction of peroxisomes induced by NUDT7 and ACOT12 deficiency also induced cellular senescence in the cartilage through the accumulation of acetyl-CoA. Lipotoxicity caused by the accumulation of fatty acids damages mitochondrial structure and function and induces a rapid increase in ROS levels, thereby contributing to cellular senescence 42. Here, exposure of iMACs to acetyl-CoA induced the SASP. However, the relevance of peroxisomal dysfunction or acetyl-CoA accumulation induced by peroxisomal dysregulation in cellular senescence has not yet been elucidated. Furthermore, the key regulatory mechanisms and molecules responsible for this process have not yet been fully investigated. Here, we found that in chondrocytes, acetyl-CoA accumulated due to a deficiency of NUDT7 and ACOT12 induces cellular senescence via upregulation of *FoxM1*, a member of the forkhead superfamily of transcription factors. FOXM1 is a crucial regulator of many biological responses and dysregulation of FOXM1 is known to significantly contribute tumorigenesis and cancer progression via modulation of cell proliferation, invasion and metastasis, cancer stem cell properties, genomic instability, and cellular metabolism 43. In particular, FOXM1 controls a network of genes necessary for the G2-M transition during cell division, suggesting its involvement in cell proliferation 44. FOXM1 is overexpressed in various human malignancies, including prostate, breast, lung, colon, stomach, liver, and kidney cancer, via the activation of Ras and cyclin D1 and FOXM1-depletion is sufficient to decrease carcinogenesis by stimulating apoptosis 45-47. Unlike in cancer cells, FOXM1 is known to have the opposite effect in chondrocytes. The level of FOXM1 is significantly upregulated in the articular tissues of patients with OA, as well as in chondrocytes after stimulation with IL-1β, a key regulator of cartilage destruction in OA 48. In contrast to cancer cells, the knockdown of *FoxM1* in IL-1 β-induced chondrocytes improved cell viability and attenuated inflammatory responses. In this study, we found that the upregulation of FOXM1 induced by a deficiency of NUDT7 and ACOT12 was responsible for the stimulation of chondrocyte senescence during OA pathogenesis. Taken together, in this study, we found that peroxisomal dysfunction induced by NUDT7 and ACOT12 deficiency stimulated cartilage degradation by activating chondrocyte senescence through the upregulation of FOXM1 via acetyl-CoA accumulation. Furthermore, the implantation of MnO_2_ nanosheets to scavenge ROS generated by senescent chondrocytes or delivery of *siFoxM1* conjugated with ACBP significantly suppressed chondrocyte senescence and cartilage degradation during OA pathogenesis.

## Materials and Methods

### Ethical approval

All animal studies were approved by Wonkwang University Animal Care and Use Committee and performed in compliance with the institutional guidelines (#WKU21-36, #WKU22-40, #WKU22-41, #WKU22-42). Human articular cartilage specimens were obtained from patients who underwent total knee arthroplasty. Human cartilage tissue collection was approved by the Human Subjects Committee of Wonkwang University Hospital, and the studies were performed in compliance with the institutional guidelines (WKUH 201605-HRBR-041). Written informed consent was obtained from all adult patients or at least one guardian of each patient before the start of the experiment.

### Human OA specimens

Human articular cartilage specimens were obtained from patients undergoing total knee replacement surgery and classified into relatively healthy (non-OA) and severely damaged (OA) regions. Human articular cartilage specimens were cut into pieces and some pieces were immediately fixed in 10% neutral buffered formalin.

### Animals

*Nudt7^-/-^* and *Acot12^-/-^* mice were generated using TALEN- or RGEN-induced mutations, as previously described 25,26. dKO mice were generated by crossbreeding of *Nudt7^-/-^* mice with *Acot12^-/-^* mice. Wild-type C57BL/6N mice were purchased from Samtako BioKorea, Inc. (Osan, Korea). All mice were housed at 22 ± 1℃ with 12 h light/dark cycles and a relative humidity of 50 ± 5% with food and water available *ad libitum*. DMM was performed in eight-week-old mice and knee joints were analyzed eight weeks after surgery. The degree of cartilage degradation was scored from 0 to 6 using the Osteoarthritis Research Society International (OARSI) scoring system.

### Alizarin red/Alcian blue staining for a newborn skeleton

Postnatal 0 pups were euthanized, skinned, and eviscerated including eyes and fat. After fixation in 95% ethanol for 24 h, specimens were submerged in Alcian blue solution overnight at room temperature (RT). Specimens were destained in 95% ethanol and transferred to 2% KOH for cleaning the tissue. The mice were soaked in alizarin red staining solution for 24 h at RT to counterstain the bone. After clearing tissues in 20% glycerol/1% KOH, 50% glycerol/1% KOH, and 80% glycerol/1% KOH for 24 h each, samples were stored in 100% glycerol.

### Histological analysis

Cartilage was fixed in 10% neutral buffered formalin and decalcified in 14% EDTA (pH 7.4). The tissues were embedded in paraffin and sectioned to a thickness of 5 μm. Safranin O staining was performed after rehydration, and the Mankin's score was measured. For immunohistochemistry, antigen retrieval using 0.01 M sodium citrate buffer (pH 6.0) was performed and sections were blocked with normal horse serum. Sections were incubated overnight with the following primary antibodies: C1,2C (1:100, 50-1035, IBEX Pharmaceuticals, Canada), FOXM1 (1:100, sc-376471, Santa Cruz, USA), MMP13 (1:200, 3533, BioVision, USA), Aggrecan Neoepitope (1:200, 100-74350, NOVUS, USA), and P16INK4A (1:100, ab189034, abcam, UK). After incubation with a peroxidase-conjugated secondary antibody, detection with the ImmPACT DAB Substrate (Vector Laboratories, USA) was performed.

### iMACs culture

The iMACs were isolated from postnatal day 5 pups using a previously published method 25. iMACs were cultured with Dulbecco's modified Eagle's medium (DMEM; Gibco) with 10% fetal bovine serum (FBS; Gibco, USA) and 100 units/ml of penicillin and streptomycin (Gibco) at 37℃ supplied with 5% of CO_2_. *pcDNA-FoxM1* and/or *siFoxM1* were transfected for 24 h following with or without 5 ng/ml IL-1β for an additional 24 h. 50 μM acetyl-CoA or 25, 50, and 100 μM sodium acetate was treated for 24 h.

### Primary cell cultures

Mesenchymal cells (at a density of 2 × 10^7^ cells/ml) were derived from the limb bud of E9 to E12.5 mouse embryo and micromass cultured in Ham's F-12 medium containing 10% FBS, 100 units/ml of penicillin and streptomycin.

### Analysis of cell differentiation and pre-cartilage condensation

Alcian blue bound to sulfated glycosaminoglycans was extracted with 6 M guanidine-HCl and quantified by measuring the absorbance of the extract at 600 nm. Cultures were incubated with 100 μg/ml biotinylated peanut agglutinin (Sigma-Aldrich, USA) and visualized with the VECTASTAIN ABC and DAB substrate solution kit.

### Microarray using mouse chondrocytes

Mouse whole-transcript expression array analysis was performed using the Affymetrix GeneChip Whole Transcript PLUS reagent. cDNA was synthesized using the GeneChip Whole Transcript Amplification kit. Sense cDNA was then fragmented, biotin-labeled with TdT using the GeneChip WT Terminal labeling kit, and hybridized to the Affymetrix GeneChip Mouse 2.0 ST Array for 16 h at 45 °C. The hybridized array was washed, stained on a GeneChip Fluidics Station 450, and scanned using a GGCS3000 Scanner (Affymetrix, USA). Signal values were computed using the Affymetrix GeneChip Command Console software.

### RNA sequencing using human chondrocytes

RNA sequencing was performed using an Illumina HiSeq4000 instrument, libraries were quantified using quantitative real-time PCR (qPCR), and their quality was checked using an Agilent Technologies 2100 Bioanalyzer. Raw data were calculated as fragments per kilobase of transcript per million mapped reads (FPKM) for each sample using Cufflinks software. Data were logarithmically transformed and normalized using the quantile normalization method.

### RT-PCR

Total RNA was isolated using RNAiso Plus (Takara, Japan) and reverse transcribed using 5X RT Master Mix (Takara). Quantitative PCR was performed using AMPIGENE qPCR Green Mix Hi-ROX (Enzo, USA) on an ABI StepOnePlus instrument (Applied Biosystems, USA) and normalized to the 18S rRNA expression level. The qRT-PCR primer sequences used in this study are listed in Supplementary Table 1.

### TUNEL assay

An *in situ* cell death detection kit (Roche, Switzerland) was used with deparaffinized cartilage sections according to the manufacturer's instructions. Images were acquired by fluorescence microscopy after nuclear staining with 4′,6-diamidino-2-phenylindole (DAPI).

### Cell apoptosis and proliferation assay

Cell apoptosis was analyzed using the Muse Annexin V & Dead Cell Kit (MCH100105, Luminex,USA), and cell proliferation was measured using the Quick Cell Proliferation Colorimetric Assay Kit (K301, Biovision) with a Muse Cell Analyzer (Merck Millipore, USA), following the manufacturer's instructions.

### SA-β-gal staining

Cellular senescence was imaged using a Senescence β-Galactosidase Staining Kit (9860, Cell Signaling Technology, USA) following the manufacturer's instructions.

### Neutral lipid staining

Fixed cells or mouse cartilage were stained with BODIPY^493/503^ (Thermo Fisher Scientific, USA) for 20 min and mounted with DAPI mounting medium (Vector Laboratories). Fluorescence images were acquired using the cartilage images.

### Preparation of chitosan-FITC/acetyl-CoA complexes

A chitosan-FITC stock solution (1 mg/ml chitosan-FITC in 0.05 N HCl) was diluted 10-fold with pH 7.4 PBS and 10 µL of acetyl-CoA (50 mM) were added. After reacting for 6 h to ensure the formation of chitosan-FITC/acetyl-CoA complexes, the final product was purified by dialysis (MWCO:12-14 kDa, SpectraPor, USA) against DDW for 1 d.

### Synthesis of manganese oxide nanoparticle-decorated polyacrylic acid (PAA-MnO_2_)

PAA (Mw: 400.000, Sigma-Aldrich) was dissolved in 50 ml dH_2_O and a solution of potassium permanganate (KMnO_4_, Sigma-Aldrich) (75 mg in 10 ml dH_2_O). Then, 45 ml of 2-(*N*-morpholino) ethanesulfonic acid (MES, Sigma-Aldrich) (0.1 M, pH 6.0) was added slowly and allowed to react at RT for 4 h until the solution color gradually changed from purple to brown. After 4 h, the solution was dialyzed (MWCO: 3.5 kDa) against dH_2_O and freeze-dried.

### Synthesis of Rhodamine 6G-loaded PAA-MnO_2_ (Rho@PAA-MnO_2_)

Fluorescence dye Rhodamine 6G was loaded onto PAA-MnO_2_ based on the weight ratio of Rhodamine 6G to PAA-MnO_2_ (0.5% and 1%). Briefly, 1 g of PAA-MnO_2_ was dissolved in 20 ml dH_2_O and 5 mg of Rhodamine 6G was added to the PAA-MnO_2_ solution. After reacting for 12 h with stirring at RT, the solution was dialyzed against dH_2_O (MWCO 1 kDa) for 6 h and freeze-dried to obtain Rho@PAA-MnO_2_ (0.5%).

### Synthesis of gallic acid-grafted chitosan oligosaccharide (COS-GA)

COS-GAs were synthesized using an EDC coupling agent. Briefly, 1 g of COS (Mn 5 kDa, TCI, Japan) was dissolved in 100 ml dH_2_O. Then, 0.49 g of GA (Sigma-Aldrich), 0.34 g of 1-(3-Dimethylaminopropyl)-3-ethylcarbodiimide hydrochloride (EDC, Tokyo, Japan), and 0.34 g of *N*-hydroxysuccinimide (NHS, Sigma-Aldrich) were added dropwise to the COS solution and reacted for 12 h at a pH of 5.0. The product was dialyzed against HCl solution (pH 2; MWCO: 1 kDa) for 4 h and freeze-dried.

### Preparation and characterization of COS-GA/*siFoxM1* complexes

Polyelectrolyte complexes of *siFoxM1* were formed using COS-GAs. Briefly, 4.5 mg of COS-GA was dissolved in 724 μl of PBS buffer (pH 7.4), and 50 μl of 20 nM *siFoxM1* solution was prepared as a stock solution. *siFoxM1* was complexed with COS-GA at an N/P ratio of 10 at 4°C for 15 min.

### Preparation and characterization of COS-GA/heparin/ACBP complexes

To prepare the COS-GA/heparin/ACBP complexes, 1 μl of 2 μg/μl Acyl-CoA-binding protein (MyBioSource, USA) and 2 μl of 2.5 mg/ml heparin (Sigma-Aldrich) were mixed for 30 min. Then, 2 μl of COS-GA solution was added and the resulting solution reacted for 3 h.

### Statistical analyses

Data are presented as the means ± SD from at least three experiments. The results were analyzed using the Student's t-test and one- or two-way analysis of variance (ANOVA) using GraphPad Prism. Significance was defined as * P < 0.05, ** P < 0.01, ***P < 0.001, and ****P < 0.0001.

## Supplementary Material

Supplementary figures and table.
