# Supplementary material for: Upregulated FOXM1 stimulates chondrocyte senescence in Acot12-/-Nudt7-/- double knockout mice
Source: Theranostics. 2023 Sep 25;13(15):5207–22. doi: 10.7150/thno.89033 (PMC10614692; doi:10.7150/thno.89033)
Supplement: Supplementary file 1 — Supplementary figures and table. [file thnov13p5207s1.pdf]

Suppl Figure 1. **Association and genotyping of *Nudt7* and *Acot12* knockout mice. (A)** Correlation between Nudt7 and Acot12 regulating β-oxidation and coenzyme A homeostasis. **(B)** Genotyping of *Nudt7*$^{-/-}$ *Acot12*$^{-/-}$ dKO mice.

Suppl Figure 2. **H&E staining of tibialis anterior (TA) muscle in 12- and 20-month-old dKO mice and peroxisomal gene profile of TA muscle in 12- and 20-month-old dKO mice (n = 5).**

Suppl Figure 3. **Cell proliferation in WT, *Nudt7*$^{-/-}$, *Acot12*$^{-/-}$, and dKO iMACs (n = 4).**

Suppl Figure 4. ***In vitro* characteristics of WT, *Nudt7*$^{-/-}$, *Acot12*$^{-/-}$, and dKO iMACs. (A)** Cell proliferation in WT and dKO iMACs (n = 4). **(B)** Apoptotic cell death in WT and dKO iMACs (n = 3). **(C)** Mitotracker staining. Scale bar = 10 μm. **(D)** BODIPY$^{493/508}$ staining. Scale bar = 20 μm**. (E)** Oil red O staining (n = 3). Quantification of Oil red O staining. **(F)** Expression level of genes in lipid metabolism. *P < 0.05; **P < 0.01; *** P < 0.001; **** P < 0.0001.

Suppl Figure 5. **Enrichment signaling in dKO iMACs. (A)** List of overlapped genes among dKO iMACs, GSE 16464, and OA cartilage (H200-3). **(B)** Top canonical pathway in dKO iMACs compared with WT.

Suppl Figure 6. **Immunohistochemistry of FOXM1 in DMM cartilage of dKO mice introduced with *lenti-HA-Nudt7, lenti-HA-Acot12*, or both (n = 6).** *P < 0.05; **P < 0.01; *** P < 0.001.

Suppl Figure 7. **Translational level of *FoxM1* in WT and dKO mice with or without introduction of *FoxM1*.**

Suppl Figure 8. **Profile of lysosomal, mitochondrial, and peroxisomal genes. (A)** In *Nudt7*$^{-/-}$ (N7KO), *Acot12*$^{-/-}$ (A12KO), dKO (ANdKO); **(B)** In *FoxM1*-overexpressed iMACs (n = 3).

Suppl Figure 9. **Transfection efficiency of three *siFoxM1* (n = 3).**

Suppl Figure 10. **Accumulation of acetyl-CoA induces *FoxM1* and stimulates catabolic regulatory factors. (A)** Senescence gene signature with exposure of sodium acetate (100 μM and 1 mM) into iMACs. **(B)** Transcriptional level of *FoxM1* treated with sodium acetate in a dose-dependent manner (n = 3). **(C)** Expression levels of *Col2a1*, *Mmp13*, and *Adamts5* with treatment of sodium acetate (n = 3).

Suppl Figure 11. **Fluorescence intensity of Rho@PAA-MnO_2 in senescence sensor after treatment with 0.5% or 1% H_2O_2.**

Suppl Figure 12. **Senescence sensing images of WT, *Nudt7*$^{-/-}$, *Acot12*$^{-/-}$, and dKO passaged (P4 to P9) iMACs treated with 1mM H_2O_2.**

Suppl Figure 13. **Senescence signaling increases with OA progression. (A)** IVIS images of senescence sensing from DMM cartilage at one, four, six, and eight weeks after surgery. **(B)** Safranin O staining and immunohistochemistry of $P16^{INK4a}$ in DMM cartilage at one, four, six, and eight weeks after surgery.

Supplementary Fig. 1

A

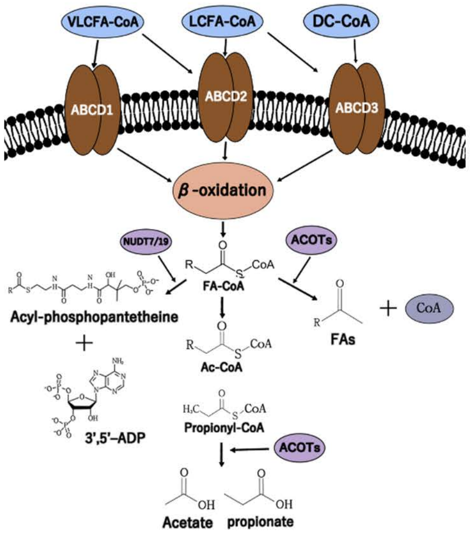

B

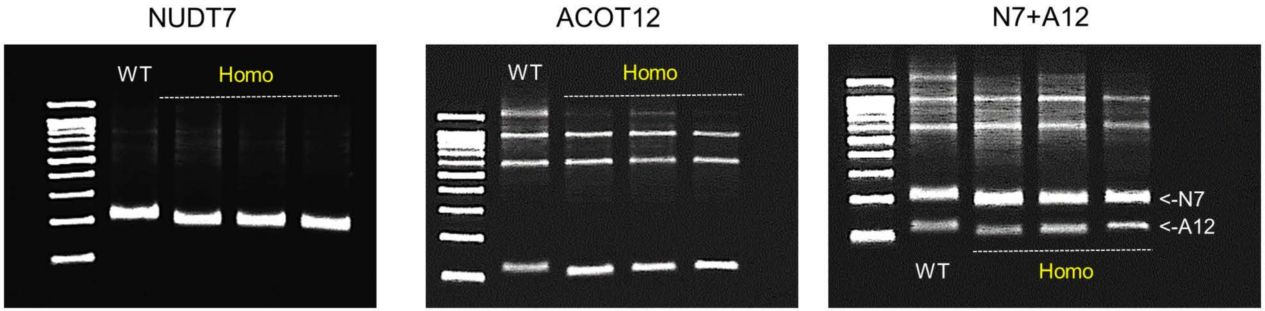

Supplementary Fig. 2

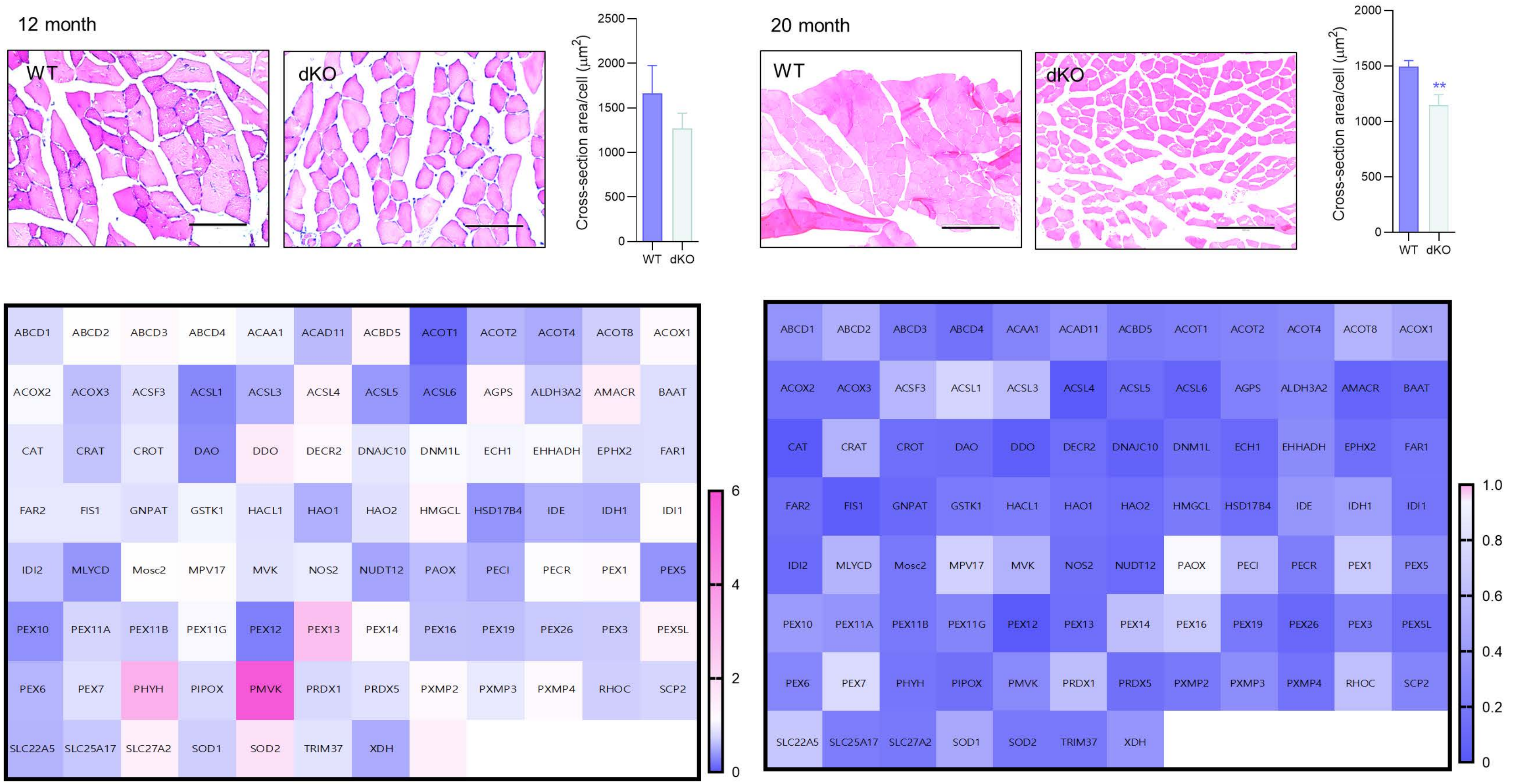

Supplementary Fig. 3

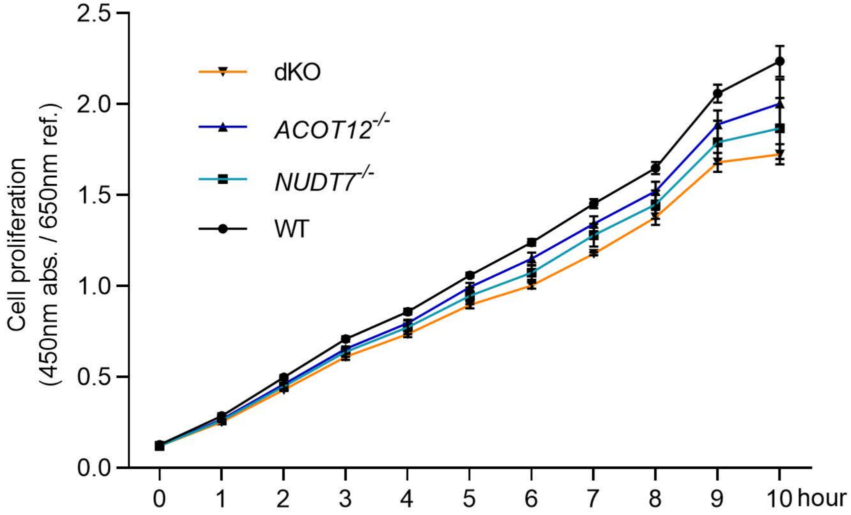

Tukey's multiple comparisons test			
Hour	WT vs. *NUDT7*-/-	WT vs. *ACOT12*-/-	WT vs. dKO
0	ns	ns	ns
1	ns	ns	ns
2	ns	ns	ns
3	ns	ns	*
4	ns	ns	**
5	**	ns	****
6	****	ns	****
7	****	*	****
8	****	**	****
9	****	****	****
10	****	****	****

Supplementary Fig. 4

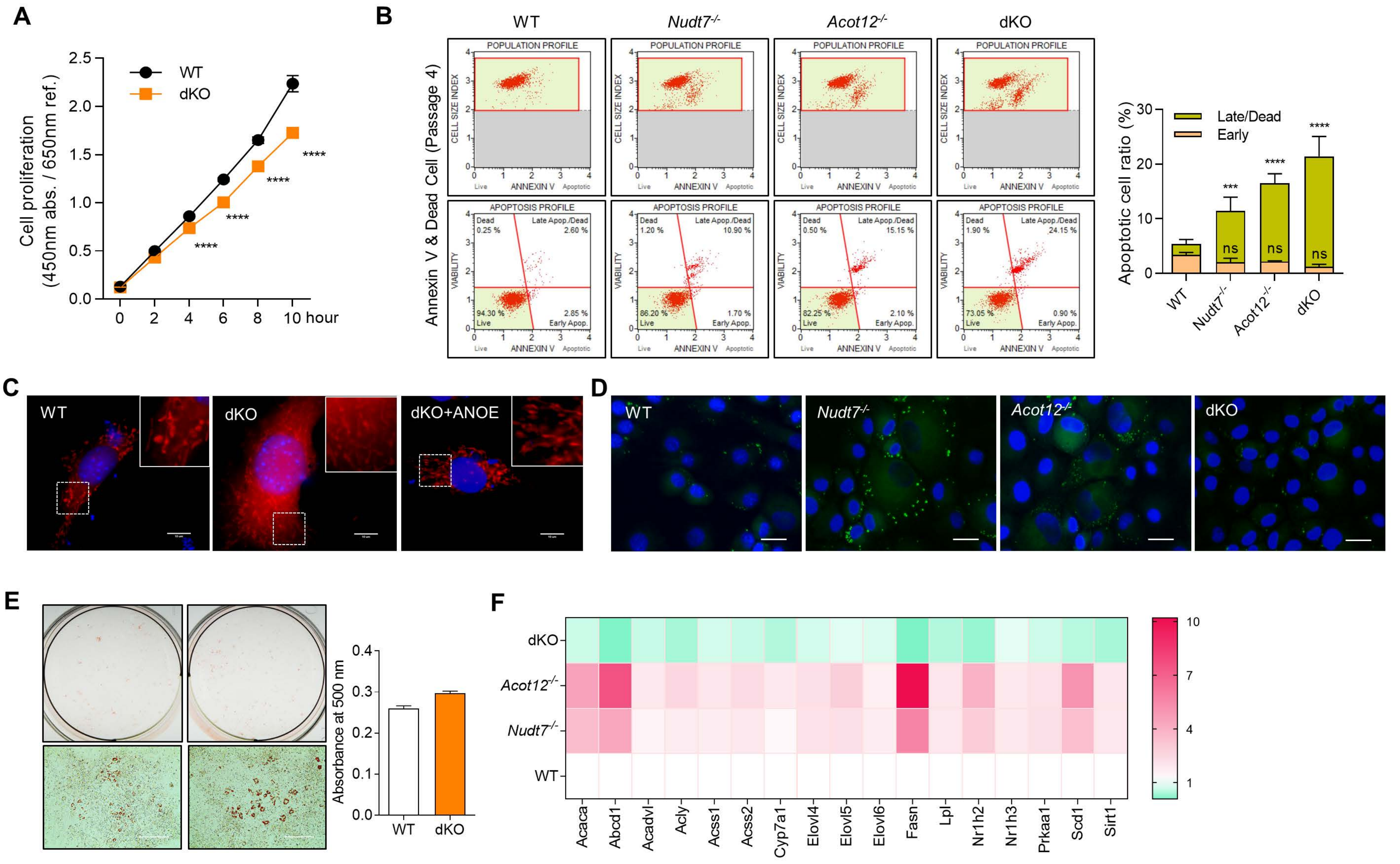

Supplementary Fig. 5

A

Upstream Regulator		dKO			GSE16464			H200-3 vs N122		
**Symbol**	**Description**	**z-score**	**p-value**	**FC**	**z-score**	**p-value**	**FC**	**z-score**	**p-value**	**FC**
let-7	microRNA	-5.529	0.000	#N/A	-3.578	0.000	#N/A	-3.592	0.000	#N/A
RB1	Retinoblastoma 1	-3.230	0.000	1.020	-2.297	0.071	1.074	-3.613	0.000	-1.005
TCF3	Transcription factor 3	-3.665	0.000	-1.040	-2.254	0.084	1.000	-3.492	0.000	1.342
mir-21	microRNA	-2.291	0.000	#N/A	-2.505	0.000	#N/A	-2.422	0.000	#N/A
NPPB	Natriuretic peptide B	-2.213	0.002	1.130	-2.213	0.104	-1.080	-2.258	0.000	1.019
HGF	Hepatocyte growth facotr	4.445	0.000	1.290	5.375	0.000	-1.026	3.251	0.000	-1.121
PTGER2	Prostaglandin E receptor 2	5.673	0.000	-1.070	3.081	0.156	1.184	4.493	0.000	-2.096
FOXM1	Forkhead box M1	5.454	0.000	3.450	2.967	0.001	1.240	4.927	0.000	2.042
ESR1	Estrogen receptor 1	6.118	0.000	-1.170	3.110	0.000	-1.211	6.609	0.000	-1.001

B

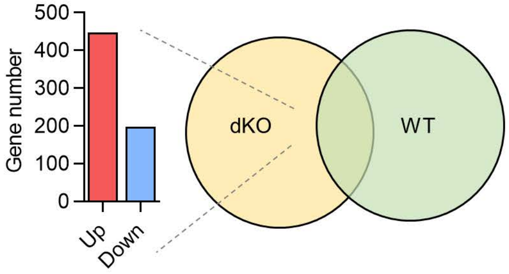

Top Canonical pathway

	p-value
Cell cycle: G2M DNA Damage Checkpoint Regulation	1.6E-08
ATM signaling	7.31E-07
Cell Cycle Control of Chromosomal Replication	7.7E-07

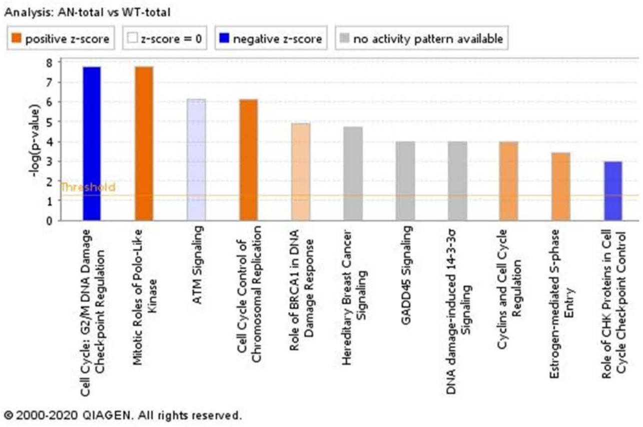

Supplementary Fig. 6

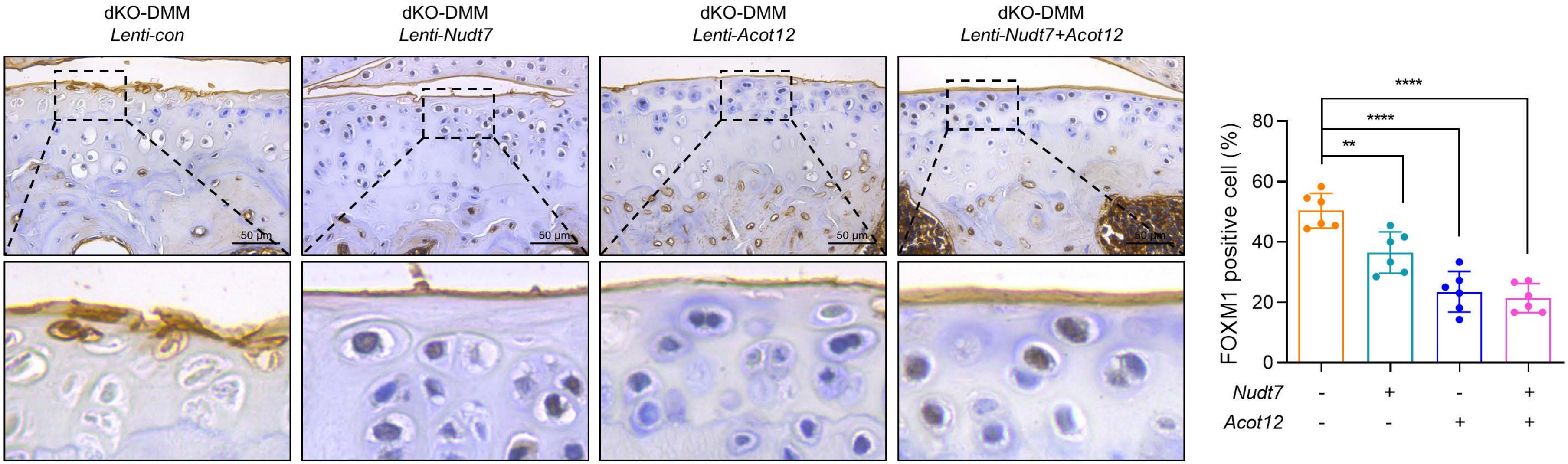

Supplementary Fig. 7

Western blot
Sample : WT, dKO-con, FoxM1 OE
EV : pcDNA empty vector
WB antibody : FOXM1

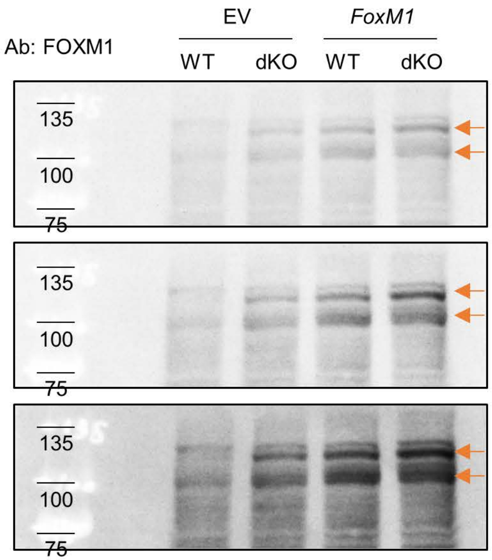

Supplementary Fig. 8

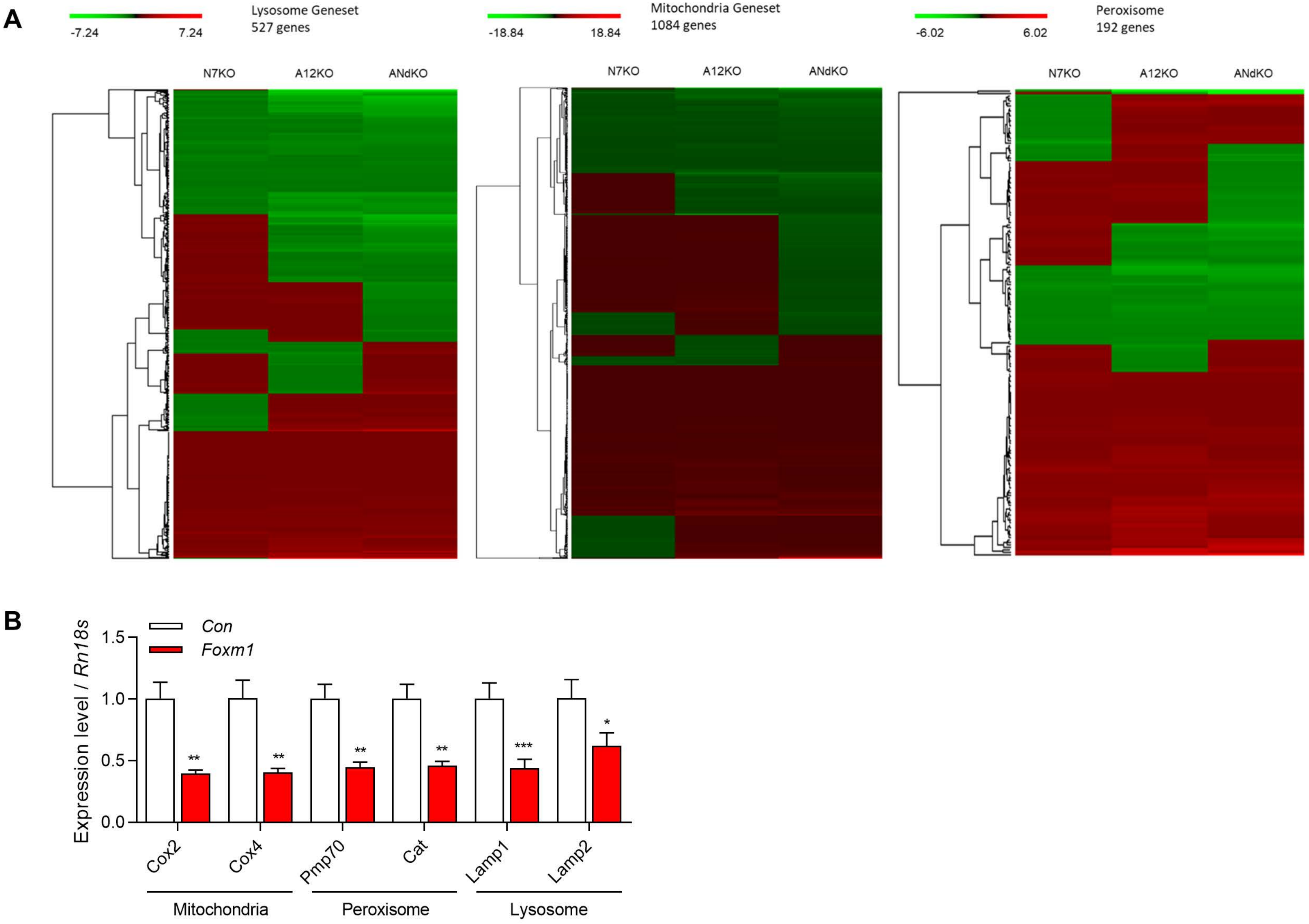

Supplementary Fig. 9

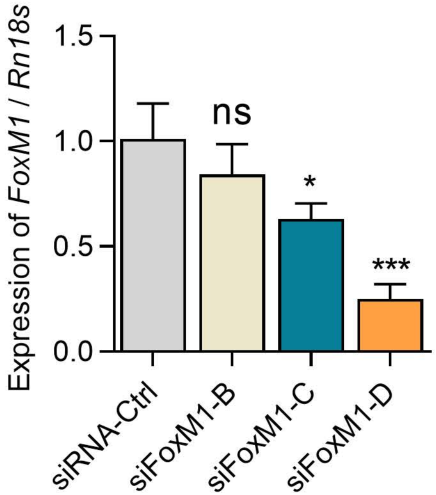

Supplementary Fig. 10

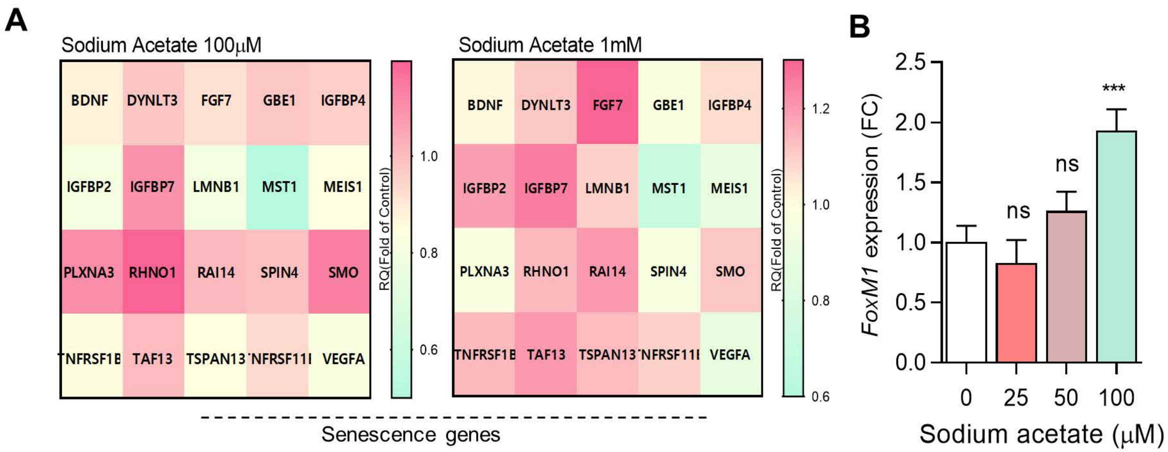

Supplementary Fig. 11

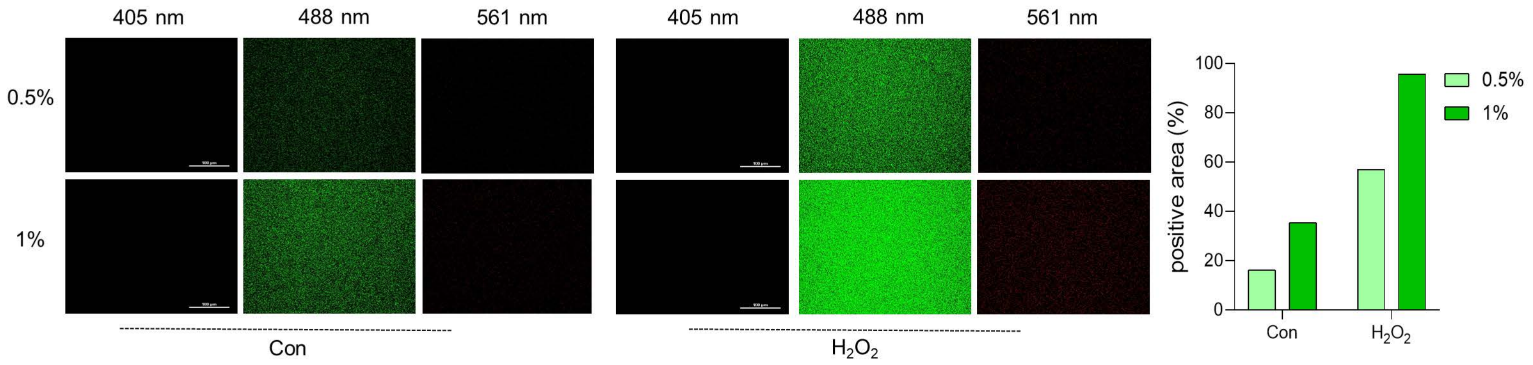

Supplementary Fig. 12

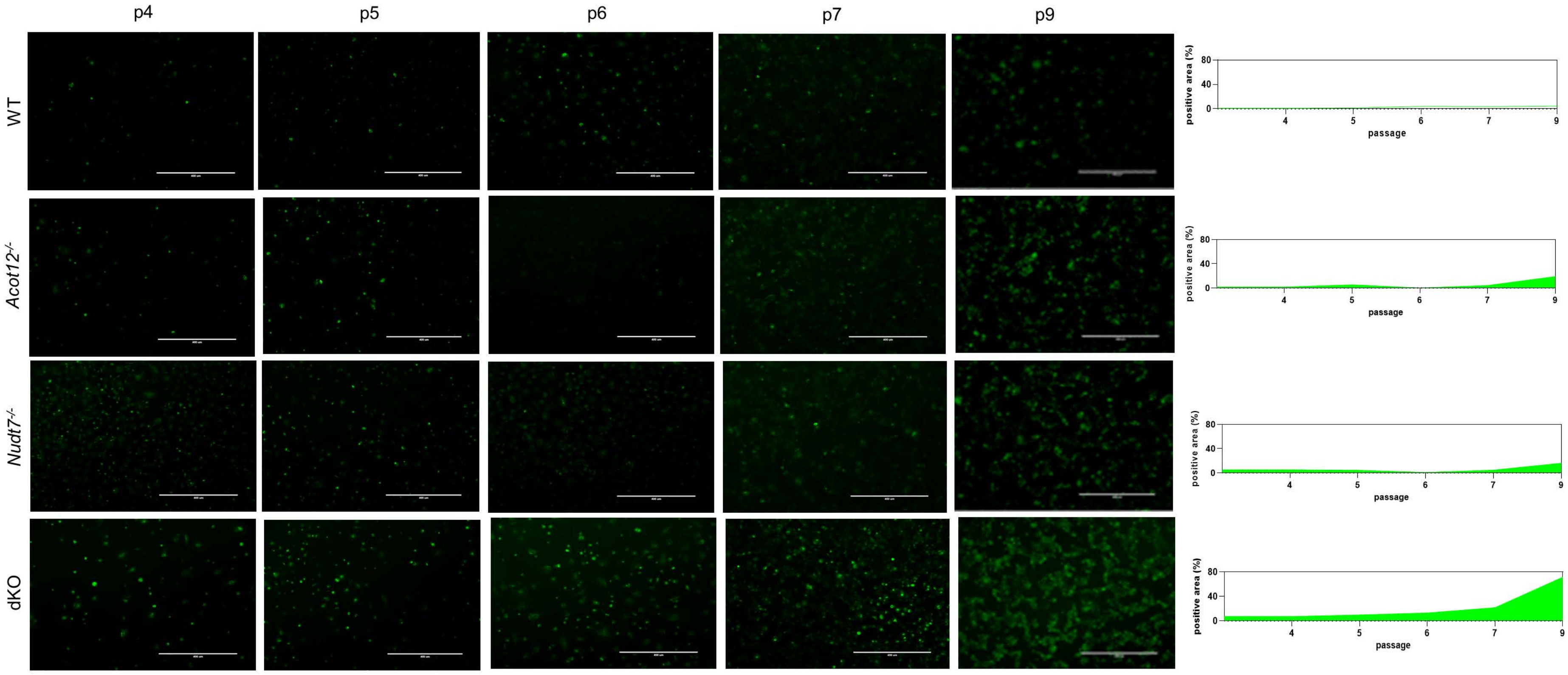

Supplementary Fig. 13

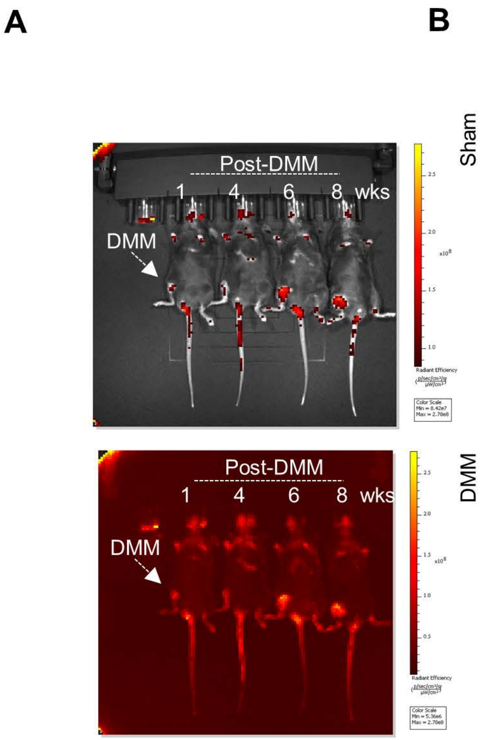

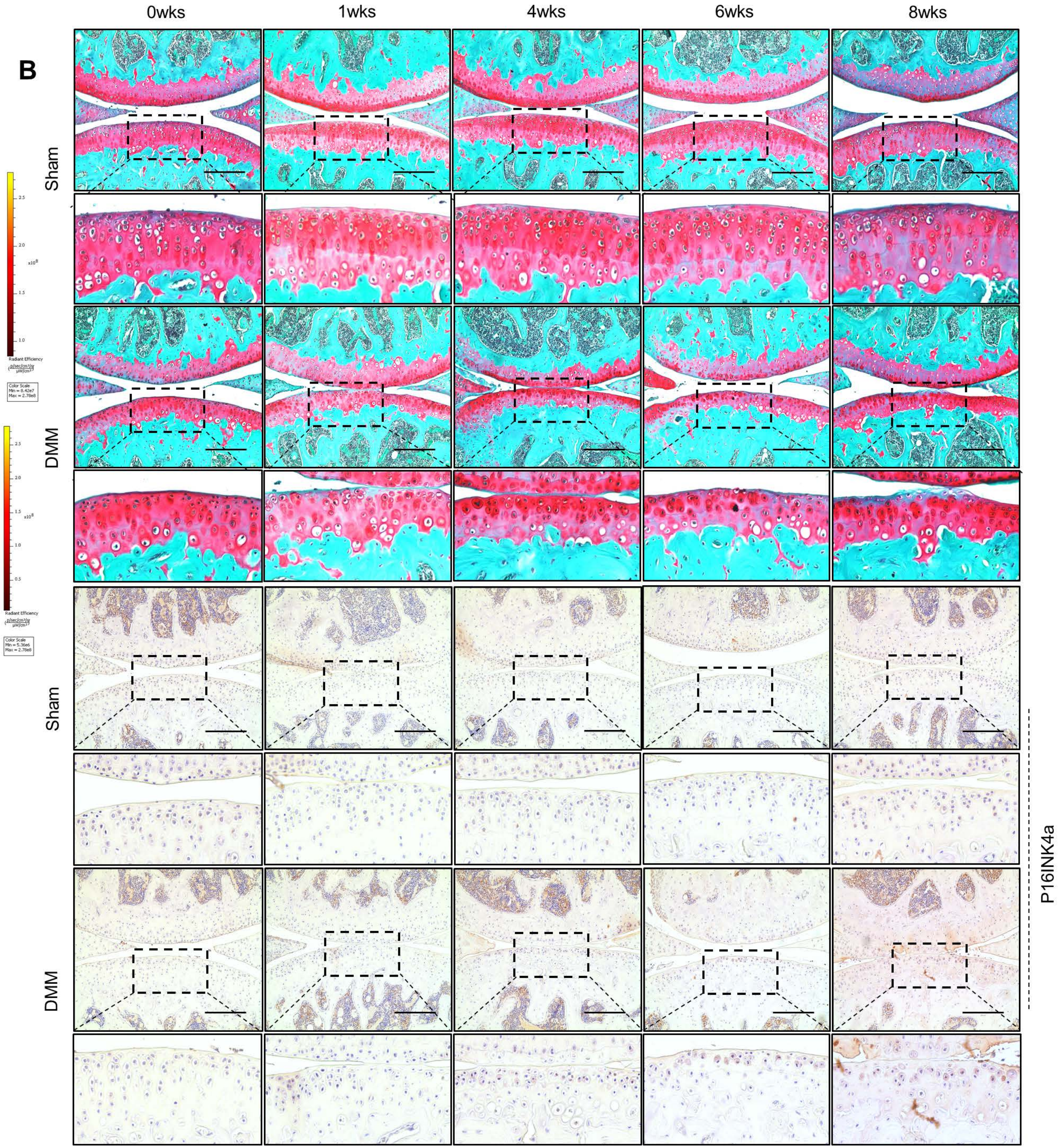

Supplementary Table 1. qRT-PCR primer sequences

Gene	Forward primer	Reverse primer
RN18S	CCAGTAAGTGCGGGTCATAAG	GGCCTCACTAAACCATCCAA
Acot12	CTCAGTGCACAGTGGGATATAAG	CTTCTCCAAGGTGGTGGTAATC
Nudt7	GCCAGAGGAGGAAAGTTGTATT	GTGGCTGTGTCATCTGTGT
Col2a1	CTGGTTTGGAGAGACCATGAA	GAGGAAAGTCATCTGGACGTTAG
Acan	GAGACTTCTGCCTCTGGAATAG	CTCCAGAAGGAATCCCACTAAC
Comp	CGTGGGCTGGAAGGATAAA	TACTAGCTCAGGACCCTCATAG
Mmp3	GGACCAGGGATTAATGGAGATG	TGAGCAGCAACCAGGAATAG
Mmp9	TCTGTATGGTCGTGGCTCTAA	GGAGGTATAGTGGGACAC
Mmp13	CCCTGATGTTTCCCATCTATACC	TTCATCGCCTGGACCATAAAG
Adamts4	GCATTCCATGGTACAGGGTTA	AGTTGACAGGGTTTCGGATG
Adamts5	TGCCACAGACCCAACTAAAG	CCATGGCTGATGACAGAGTT
IL-6	GGGTTGCTTCTCTGTGTCTT	GGTCGTCTTGCTTTCCTTCT
TNFa	TTGTCTACTCCCAGGTTCTCT	GAGGTTGACTTTCTCCTGGTATG
Ccl4	CCACTTCCTGCTGTTTCTCTTA	CAAAGACTGCTGGTCTCATAGT
Ccl5	GCCCACGTCAAGGAGTATTT	CTTGAACCCACTTCTTCTCTGG
IL-1b	CCACCTCAATGGACAGAATATCA	CCCAAGGCCACAGGTATTT
Casp3	CAGTGGACTCTGGGATCTATCT	TGACATTCCAGTGCTCTTATGG
Casp9	AGACCTTGGATGGCATTCTG	CAGCCAGGAATCTGCTTGTA
Casp1	ACAAGGCACGGGACCTATG	TCCCAGTCAGTCCTGGAAATG
ATG3	GCTGGAGGTGAAGATGCTATTT	TAGCCGTGGTGTCTGGTAATA
ATG12	GCCTCGGAACAGTTGTTTATTT	CAGGACCAGTTTACCATCACT
JNK	TCCAGCACCCATACATCAAC	CTATTGTGTGCTCCCTCTCATC
FAS	AGACAGGATGACCCTGAATCTA	CTTCAGCAATTCTCGGGATGTA
Nf-kB	GGATGACAGAGGCGTGTATTAG	CCTTCTCTCTGTCTGTGAGTTG
Acaca	AGCCAGAAGGGACAGTAGAA	CTCAGCCAAGCGGATGTAAA
Abcd1	GTACAGTCTGGTGTCCATGTAG	GGGTGTGATGATAGGGATGTT
Acadvl	CTTTGCAGGGACTCAAGGAA	CAAGCGAGCATACTGGGTATTA
Acly	CGGGAGGAAGCTGATGAATATG	GTCAAGGTAGTGCCCAATGAA
Acss1	GAGCATGAGCAGTGAAGACA	CATGGCGGCATACAGTAGATAG
Acss2	CACCTTCTGGCAAACAGAAAC	CTACACCGAAGAATGGGAAAGA
Cyp7a1	CACCTTGAGGATGGTTCCTATAA	TCAAAGGGTCTGGGTAGATTTC
Elovl4	GACCTGGACCATTGCAGATAA	GCCACACGAACAGGAGATAG
Elovl5	GGTGTGTGGGAAGGCAAATAC	TGGAGAAGTAGTACCACCAGAG
Elovl6	CCGAACTAGGTGACACGATATT	TGTAGGAGTACCAGGAGTACAG
Fasn	AAGTTGCCCGAGTCAGAGAA	TTCCAGACCGCTTGGGTAAT
Lpl	GCCCGAGGTTTCCACAAATA	GCTGAAGTAGGAGTCGCTTATC
Nr1h2	CCACGTCACCCACTATTAAGG	GCTCTAAGATGACCACGATGTAG
Nr1h3	GTGGAAGACAGAACCTCAAGAT	TGACTCCAACCCTATCCCTAA
Prkaa1	GATCCTTTCCGGTGTGGATTAT	GAAAGACCAAAGTCGGCTATCT
Scd1	CAACTTCACCACGTTCTTCATC	CCCGTCTCCAGTTCTCTTAATC
Sirt1	GGAACCTTTGCCTCATCTACA	CACCTAGCCTATGACACAACTC

Acox1	CGCACATCTTGGATGGTAGT	GGCTTCGAGTGAGGAAGTTATAG
Fabp4	GGATGGAAAGTCGACCACAATA	TGGCTCATGCCCTTTCATAA
Cd36	CTGGGACCATTGGTGATGAAA	CACCACTCCAATCCCAAGTAAG
BDNF	GGTCACAGTCCTAGAGAAAG	CAGCCTTCCTTGGTGTAA
BMP6	AGGACTGGATCATTGCAC	GTTGGTGGCATTCATGTG
CCL2	CTCAGCCAGATGCAGTTA	GTGATCCTCTTGTAGCTCTC
CDKN1A	AAGTGTGCCGTTGTCTCTTC	AGTCAAAGTTCCACCGTTCTC
CXCL1	GCTGGGATTCACCTCAAGAA	TGGCTATGACTTCGGTTTGG
CXCL2	GACAGAAGTCATAGCCACTCTC	GCCTTGCCTTTGTTCAGTATC
CXCL3	CAGAAGTCATAGCCACTCTC	CTTGCCGCTCTTCAGTAT
FGF2	TACCGGTCACGGAAATAC	GAAGAAACAGTATGGCCTTC
FGF7	GCGACACACCAGAAGTTA	CTGGGTCCCTTTCACTTT
ICAM1	GTACTGCTGGTCATTGTG	CCTGAGCCTTCTGTAACT
IGFBP2	AAGTCAGGCATGAAGGAG	TCCAGACTGAGGTGTTTG
IGFBP4	CGAACATCCCAACAACAG	CATCTTGCTCCGATCTCTA
IGFBP7	GTGCTGGTATCTCCTCTAAG	GGGCATCAACCACTGTAA
LMNB1	GGGCGTCAGATTGAGTAT	CTCCAGCTCTTCCTTGTA
MMP10	GTGGTGTTCCTGATGTTG	ATCCACACTCTGTCTTGG
MST1	CTTCCACTACAACATGAGC	CACAGACTCGAGTGGTATAG
TNFRSF11B	GAAACCCTTCCTCCAAAGTA	CAATGTCTTCCTCCTCACTG
TNFRSF1B	CTGTAGCATCCTGGCTATTC	TCTGGCTGAGATACGTAGAG
VEGFA	CTTTACTGCTGTACCTCCAC	CTGGTAGACATCCATGAACT
MEIS1	CCCTCTCTTAGCACTGATTT	CATTGAATGACTCTGACGAG
RHNO1	TTTGAGAGTCCACAGTCTTC	CTGAACAATGGCACTAAAGG
SMO	CCCATCAAGATCCATTTCTC	AAGATCTCAGCCTCCATTAG
SPIN4	CCCACTTTCCTCACTCATAG	GAGCACAGTACCTTTCCATT
ZC3H4	AGACCGTCAATGTCAATACC	ATCTGCTGGCTTCTGTAATC
CCND1	CAGAGGCGGATGAGAACAAG	GAGGGTGGGTTGGAAATGAA
DYNLT3	CAAGCATAGTGGAACAGTCT	CTGGCTGTGTGAAATCCATA
GBE1	CAAGAGCTATACGGACTACC	GTCTCTGATGACCTCCATAC
GDNF	CTGACCAGTGACTCCAATATG	GCTTGTTTATCTGGTGACCT
P4HA2	AAGCTCGCCAAGATTAAGAG	GTAGGCATTCACAGGATGAG
PLK3	GTCTGTTTGCCAAAGTTACC	ACCAAGCAGGAGACATTATC
PLXNA3	CCTTCTCCCATTGCTATTC	GTGAGTCAGAGTGGTATCT
RAI14	AAGATTCTTCCGGTCACAG	CCGAGTTGTCAATGTTCTC
SLC16A3	CTGCAGAAGCATTATCCAGA	ATTGAGCATGATGAGGGAAG
TAF13	CTGAAATGACTCACAAGGC	CTCTAGCAAACTTCCTTGG
TMEM87B	GACAAATCAGGACCTTTGG	GCCACTCTATGGTAAACTTC
TSPAN13	CTTGTTTAGCTCTGAATCGG	GCAGTTCAAATTCCTCTGG
UFM1	GCTAAAGTTTGCAGCAGAAG	ACATTCCCAGCAGTCTGT
FOXM1	AGGAGGACATAGCACACTTAGA	GGAGTTGGATAGCTCCTCTTTG
Cox2	CATCCCAGGCCGACTAAAT	TGGGCATAAAGCTATGGTTAGA
Cox4	AGTTGTACCGCATCCAGTTT	GCAGTGAAGCCAATGAAGAAC

Pmp70	CTTGCTTGTTTCTGGGCTATTC	CGTATCTGTATTCTCCTTCGTACTT
Cat	GATGGTAACTGGGATCTTGTGG	GTGGGTTTCTCTTCTGGCTATG
Lamp1	GACCCTGAAAGTGGAGAACAA	GGGCATCAGGAAGAGTCATATT
Lamp2	CTGACTCCTGTCGTTCAGAAAT	GGTGGGAGTTTGGTCTTCTT
Erfdh	AGCTACACGCTGTTCGAAATA	GGCTCCATTCCTCTCAATATCC
Etfb	GTGGTCACTGATGGTGTGAA	CTCCTTCACCAGTTTCTTCTCC
Decr1	CTGGGAACTATGGAGGAACTTG	CTTCCTCTCCACCGTCAAATC
Hadha	AGACATCGGAGCTGTCTTTG	CACTACCTTCTGAGCACCATAC
Hadhb	CCAAGAAGGCACAGGATGAA	AGGAAGGACGGATCCCATTA
Hadh	CTTGACTATGTTGGACTGGATACT	GGAAGGACTGGGCTGAAATAA
Acaa2	GACTTCTCTGCCACCGATTTA	TTGCCCACGATGACACTATC
Cpt2	CTCAGAAATCCAGGCACATCTA	CTCGGTTCTCACTGGTCAAATA
Acadm	GAGAAGAAGGGTGACGAGTATG	GGCTTTACTAGCGGGTACTTTA
Acads	CCCACAGCTCAGGTTAAGAA	GATGGAGTAGGCCAGGTAATC
